# Supplementary material for: Antepartum and labour-related single predictors of non-participation, dropout and lost to follow up in a randomised controlled trial comparing internet-based cognitive–behaviour therapy with treatment as usual for women with negative birth experiences and/or post-traumatic stress following childbirth
Source: BMJ Open. 2022 Nov 28;12(11):e063214. doi: 10.1136/bmjopen-2022-063214 (PMC9710351; doi:10.1136/bmjopen-2022-063214)
Supplement: Supplementary data [file bmjopen-2022-063214supp001.pdf]

Supplementary Table 1.

Week by week content of the iCBT treatment

Week	Content
1st	Information, psychoeducation, breathing retraining
2nd	Vignettes, common symptoms, fear and avoidance
3rd	Depressive symptoms, significance of relationships, “reflective listening”
4th	Exposure, talking about the childbirth
5th	Managing anxiety and depressive symptoms, psychological health, values, recovery
6th	Summary, repetition and relapse prevention

Note. Every week contained homework assignments based on the content of the module
