# Supplementary material for: Antepartum and labour-related single predictors of non-participation, dropout and lost to follow up in a randomised controlled trial comparing internet-based cognitive–behaviour therapy with treatment as usual for women with negative birth experiences and/or post-traumatic stress following childbirth
Source: BMJ Open. 2022 Nov 28;12(11):e063214. doi: 10.1136/bmjopen-2022-063214 (PMC9710351; doi:10.1136/bmjopen-2022-063214)
Supplement: Supplementary data [file bmjopen-2022-063214supp002.pdf]

Supplementary Table 2.
Odds ratio with 95% CI, Beta values, and SE derived from logistic regression, for potential predictors

	Non-participants		Pre-treatment dropout		Treatment dropout		Lost to follow up	
	N	OR 95% CI<br>*B*, S.E	N	OR 95% CI<br>*B*, S.E	N	OR 95% CI<br>*B*, S.E	N	OR 95% CI<br>*B*, S.E
Country of birth<br>Sweden/other	1234<br>953/281	3.93(2.58-6.15)***<br>1.38, 0.22	284<br>259/25	1.09(0.45-2.64)<br>0.09, 0.45	98<br>89/9	1.43 (0.33-6.06)<br>0.35, 0.74	198<br>181/17	1.87(0.69-5.08)<br>0.63, 0.51
Level of education	1336		290		99		199	
University	775	1.0	208	1.0	71	1.0	148	1.0
High school	489	1.83(1.38-2.44)***<br>0.61, 0.15	81	1.53(0.89-2.62)<br>0.43, 0.28	27	1.32(0.53-3.28)<br>0.28, 0.46	50	1.33(0.69-2.55)<br>0.28, 0.33
Elementary school	72	26.2(3.62-189.9)***<br>3.27, 1.01	1	na	1	na	1	na
Relationship status<br>married-cohabit/other	1357<br>1291/66	2.06(0.97-4.37)<br>0.72, 0.38	292	1.25(0.29-5.35)<br>0.256, 0.74	97<br>95/2	na	197<br>192/5	na
Age<br>years	1510	0.99(0.96-1.01)<br>-0.12, 0.013	300	0.97(0.92-1.02)<br>-0.034, 0.86	99	0.95(0.86-1.05)<br>-0.52, 0.05	199	0.95(0.89-1.02)<br>-0.048, 0.03
Previous CS<br>no / yes	1491<br>1302/189	0.80(0.53-1.19)<br>-0.23, 0.21	295<br>31/264	0.78(0.36-1.68)<br>-0.25, 0.39	98<br>89/9	1.42(0.33-6.06)<br>0.35, 0.74	196<br>177/19	0.87(0.33-2.32)<br>-0.14, 0.50
Counselling for fear of childbirth, no / yes	1523<br>1376/147	0.50(0.35-0.73)***<br>-0.69, 0.19	300<br>254/46	1.06(0.55-2.05)<br>0.06, 034	99<br>87/12	0.68(0.18-2.52)<br>-0.40, 0.62	199<br>169/30	0.88(0.39-1.97)<br>-0.13, 0.41
Preeclampsia<br>no / yes	1523<br>1440/83	0.59(0.36-0.96)*<br>-0.53, 0.25	300<br>276/24	1.20(0.51-2.85)<br>0.18, 0.44	99<br>90/9	1.46(0.34-6.22)<br>0.38, 0.74	199<br>184/15	1.39(0.48-4.00)<br>0.33, 0.54
Length of pregnancy<br>days	1507	1.00(0.99-1.00)<br>-0.003, 0.004	299	0.99(0.98-1.01)<br>-0.008, 0.008	99	1.01(0.98-1.04)<br>0.01, 0.013	198	1.00(0.98-1.02)<br>0.002, 0.01
Parity	1505		299		99		198	
1st child	838	1	200	1	68	1	134	1

2nd child	448	1.65(1.22-2.22)**<br>0.50, 0.15	71	0.97(0.55-1.73)<br>-0.28, 029	22	1.80(0.65-4.96)<br>0.58, 0.52	48	1.07(0.54-2.10)<br>0.06, 0.34
3rd child or more	219	2.15(1.40-3.30)***<br>0.77, 0.081	28	1.52(0.68-3.40)<br>0.42, 0.41	9	1.68(0.39-7.26)<br>0.52, 0.75	16	1.63(0.58-4.60)<br>0.49, 0.53
Mode of delivery	1523		300		99		199	
Vaginal delivery	783	1	129	1	40	1	82	1
Emergency CS	289	0.71(0.51-1.0)<br>-0.34, 0.17	63	1.0(0.54-1.88)<br>0.003, 0.32	20	0.33(0.11-1.03)<br>-1.1, 0.58	40	0.74(0.34-1.58)<br>-0.31, 0.39
Immediate CS[1]	186	0.54(0.37-0.79)**<br>-0.61, 0.19	49	0.63(0.30-1.30)<br>-0.46, 0.37	19	0.19(0.06-0.63)**<br>-1.64, 0.60	36	0.42(0.18-0.99)*<br>-0.86, 0.43
Vacuum assisted	198	0.62(0.43-0.91)*<br>-0.475, 0.19	48	0.96(0.48-1.91)<br>-0.04, 0.35	15	0.29(0.84-1.01)<br>-1.23, 0.63	31	0.26(0.10-0.71)**<br>-1.33, 0.51
Elective CS	67	1.01(0.52-1.99)<br>0.01, 0.34	11	0.17(0.02-1.41)<br>-1.75, 1.06	5	1.33(0.13-13.37)<br>0.29, 1.18	10	2.57(0.62-10.65)<br>0.95, 0.73
Foetal presentation	1510	1.02(0.71-1.46)	300	0.53(0.25-1.13)	99	0.31(0.11-0.94)*	199	1.28(0.61-2.70)
Vertex / other	1287/223	0.20, 0.18	256/44	-0.63, 0.38	82/17	-1.16, 0.56	165/34	0.24, 0.38
Manual placenta removal<br>no / yes	1523<br>1379/144	1.41(0.88-2.26)<br>0.346, 0.24	300<br>278/22	0.42(0.14-1.26)<br>-0.88, 0.57	99<br>93/6	0.33(0.06-1.90)<br>-1.11, 0.89	199<br>181/18	0.99(0.36-2.66)<br>-0.014, 0.51
Epidural anaesthesia<br>no / yes	1523<br>813/710	0.86(0.67-1.10)<br>-0.15, 0.13	300<br>151/149	1.12(0.69-1.80)<br>0.11, 024	99<br>49/50	0.95(0.43-2.12)<br>-0.049, 0.41	199<br>102/97	1.52(0.86-2.70)<br>0.42, 0.29
Intrapartum foetal distress<br>no / yes	1523<br>1234/289	0.67(0.50-0.91)*<br>-0.39, 0.15	300<br>228/72	0.76(0.43-1.36)<br>-0.27, 0.29	99<br>71/28	0.50(0.21-1.21)<br>-0.69, 0.45	199<br>148/51	0.50(0.25-0.99)*<br>-0.70, 0.36
Anal sphincter injury<br>no / yes	1523<br>1447/76	0.48(0.29-0.79)**<br>-0.73, 0.25	300<br>275/25	0.92(0.38-2.21)<br>-0.82, 045	99<br>88/11	1.27(0.35-4.66)<br>0.24, 0.66	199<br>182/17	1.09(0.40-3.00)<br>0.09, 0.52
Labour dystocia<br>no / yes	1523<br>885/638	0.87(0.67-1.11)<br>-0.14, 0.13	300<br>166/134	1.26(0.78-2.04)<br>0.23, 0.25	99<br>55/44	0.74(0.33-1.66)<br>-0.3, 0.41	199<br>114/85	0.75(0.42-1.34)<br>-0.29, 0.30
Severe haemmorhage[1]	1523	1.19(0.80-1.76)	300	0.38(0.15-0.96)*	99	0.49(0.17-1.44)	199	1.00(0.44-2.28)

no / yes	1329/194	0.17, 0.20	266/34	-0.95, 0.47	83/16	-0.72, 0.55	171/28	0.004, 0.42
Anaemia	1523	0.85(0.61-1.18)	300	0.57(0.29-1.12)	99	0.50(0.19-1.35)	199	0.66(0.32-1.38)
no / yes	1274/249	-0.16, 0.17	246/54	-0.56, 0.35	79/20	-0.69, 0.51	158/41	-0.41, 0.37
Blood transfusion	1523	1.01(0.69-1.49)	300	0.65(0.29-1.45)	99	0.31(0.09-1.09)	199	0.53(0.21-1.32)
no / yes	1340/183	0.011, 0.20	265/35	-0.43, 0.41	87/12	-1.19, 0.65	173/26	-0.64, 0.47
Children in the pregnancy	1508	1.35(0.52-3.55)	299	0.48(0.05-4.40)	99	na	198	0.51(0.05-4.96)
1 child / 2 children	1476/32	0.30, 0.49	294/5	-0.72, 1.12	97/2		194/4	-0.68, 1.16
Child transferred to NICU	1523	0.83(0.60-1.14)	300	1.21(0.67-2.20)	99	0.73(0.28-1.91)	199	1.07(0.52-2.22)
no / yes	1255/268	-0.19, 0.16	241/59	0.20, 0.30	78/21	-0.32, 0.49	162/37	0.069, 0.37
Breastfeeding problems	1523	0.86(0.28-2.64)	300	0.65(0.07-6.36)	99	na	199	0.77(0.07-8.67)
no / yes	1505/18	-0.15, 0.57	296/54	-0.43, 1.16	98/1		196/3	-0.26, 1.23
Overall birth experience[1]	1203	1.02(0.74-1.42)	234	0.72(0.38-1.35)	72	0.27(0.09-0.79)*	148	0.90(0.43-1.88)
0-2 / 3-5	305/898	0.023, 0.17	58/176	-0.34, 0.32	20/52	-1.31, 0.55	40/108	-0.11, 0.38

Note. The first category is the reference, for e.g. when yes/no is stated, yes is the reference category.
[1] inclusion criteria.
* p<.05, **p<.01, ***p<.001
