# Supplementary material for: Antepartum and labour-related single predictors of non-participation, dropout and lost to follow up in a randomised controlled trial comparing internet-based cognitive–behaviour therapy with treatment as usual for women with negative birth experiences and/or post-traumatic stress following childbirth
Source: BMJ Open. 2022 Nov 28;12(11):e063214. doi: 10.1136/bmjopen-2022-063214 (PMC9710351; doi:10.1136/bmjopen-2022-063214)
Supplement: Author's manuscript [file bmjopen-2022-063214.draft_revisions.pdf]

BMJ Open is committed to open peer review. As part of this commitment we make the peer review history of every article we publish publicly available.

When an article is published we post the peer reviewers' comments and the authors' responses online. We also post the versions of the paper that were used during peer review. These are the versions that the peer review comments apply to.

The versions of the paper that follow are the versions that were submitted during the peer review process. They are not the versions of record or the final published versions. They should not be cited or distributed as the published version of this manuscript.

BMJ Open is an open access journal and the full, final, typeset and author-corrected version of record of the manuscript is available on our site with no access controls, subscription charges or pay-per-view fees (http://bmjopen.bmj.com).

If you have any questions on BMJ Open's open peer review process please email info.bmjopen@bmj.com

BMJ Open

Antepartum and labour related predictors of non-participation, dropout, and lost to follow up in a treatment for women with negative birth experiences and/or post-traumatic stress following childbirth.

Journal:	*BMJ Open*
Manuscript ID	bmjopen-2022-063214
Article Type:	Original research
Date Submitted by the Author:	27-Mar-2022
Complete List of Authors:	Sjömark, Josefin; Uppsala Universitet, Department of Women's and Children's Health<br>Svanberg, Agneta ; Uppsala Universitet, Department of Women's and Children's Health<br>Viirman, Frida; Uppsala Universitet, Department of Women's and Children's Health<br>Larsson, Margareta; Uppsala University, Dep of Women's and Children's Health<br>Poromaa, Inger; Uppsala Universitet, Department of Women's and Children's Health<br>Skalkidou, Alkistis; Uppsala University, Department of Women's and Children's Health<br>Jonsson, Maria; Uppsala University, Department of Women's and Children's Health<br>Parling, Thomas; Uppsala University, Department of Women's and Children's Health; Karolinska Institutet & Stockholm Health Care Services Region Stockholm, clinical neuroscience
Keywords:	GYNAECOLOGY, MENTAL HEALTH, OBSTETRICS, Adult psychiatry < PSYCHIATRY, Anxiety disorders < PSYCHIATRY

SCHOLARONE™
Manuscripts

I, the Submitting Author has the right to grant and does grant on behalf of all authors of the Work (as defined in the below author licence), an exclusive licence and/or a non-exclusive licence for contributions from authors who are: i) UK Crown employees; ii) where BMJ has agreed a CC-BY licence shall apply, and/or iii) in accordance with the terms applicable for US Federal Government officers or employees acting as part of their official duties; on a worldwide, perpetual, irrevocable, royalty-free basis to BMJ Publishing Group Ltd ("BMJ") its licensees and where the relevant Journal is co-owned by BMJ to the co-owners of the Journal, to publish the Work in this journal and any other BMJ products and to exploit all rights, as set out in our licence.

The Submitting Author accepts and understands that any supply made under these terms is made by BMJ to the Submitting Author unless you are acting as an employee on behalf of your employer or a postgraduate student of an affiliated institution which is paying any applicable article publishing charge ("APC") for Open Access articles. Where the Submitting Author wishes to make the Work available on an Open Access basis (and intends to pay the relevant APC), the terms of reuse of such Open Access shall be governed by a Creative Commons licence – details of these licences and which Creative Commons licence will apply to this Work are set out in our licence referred to above.

Other than as permitted in any relevant BMJ Author's Self Archiving Policies, I confirm this Work has not been accepted for publication elsewhere, is not being considered for publication elsewhere and does not duplicate material already published. I confirm all authors consent to publication of this Work and authorise the granting of this licence.

For peer review only

Antepartum and labour related predictors of non-participation, dropout, and lost to follow up in a treatment for women with negative birth experiences and/or post-traumatic stress following childbirth.

Josefin Sjömark[1] MS; Agneta Skoog Svanberg[1] professor; Frida Viirman[1] BSc; Margareta Larsson[1] associate professor; Inger Sundström Poromaa[1] professor; Alkistis Skalkidou[1] professor; Maria Jonsson[1] associate professor; Thomas Parling[1,2] PhD

[1] Department of Women's and Children's Health, Uppsala University, SE-751 85 Uppsala, Sweden. | josefin.sjomark@kbh.uu.se ; agneta.skoog_svanberg@kbh.uu.se ; frida.viirman@kbh.uu.se ; Margareta.Larsson@kbh.uu.se ; Inger.Sundstrom@kbh.uu.se ; alkistis.skalkidou@kbh.uu.se ; maria.jonsson@kbh.uu.se ; Thomas.parling@kbh.uu.se

[2] Centre for Psychiatry Research, Department of Clinical Neuroscience, Karolinska Institutet, & Stockholm Health Care Services Region Stockholm, The Centre for Psychotherapy, Education & Research, Liljeholmstorget 7, SE-117 63 Stockholm Sweden | thomas.parling@ki.se

Trial Registration: This study is a dropout investigation of a randomized controlled trial (RCT). The RCT was registered at *anonymized* . Date for registration *anonymized*, retrospectively registered.

Word count: 3007

Acknowledgements

We would like to thank statistician Per Wikman for help with structuring the database.

For peer review only

Corresponding Author:

Thomas Parling

Department of Women's and Children's Health

Uppsala University

Dag Hammarskjölds väg 14B

SE-751 85 Uppsala

Sweden

Phone: 46 70 2058007

Email: thomas.parling@kbh.uu.se

For peer review only

Abstract

Objectives: Internet-based interventions are often hampered by high dropout rates. The number of individuals who decline to participate or drop out are reported, but reasons for dropout are not. Identification of barriers to participation and predictors of dropout may help improve the efficacy of internet-based clinical trials. The aim was to investigate a large number of possible predictors for non-participation and dropout in a randomized controlled trial for women with a negative birth experience and/or post-traumatic stress following childbirth.

Setting: A childbirth clinic at a university hospital in Sweden.

Participants: The sample included 1,523 women who gave birth between September 2013 and February 2018. All women who rated an overall negative birth experience on a Likert scale, and/or had an immediate caesarean section (CS), and/or severe postpartum haemorrhage (≥ 2,000 ml) were eligible.

Methods: Demographic, antepartum, and labour-related/postpartum predictors were investigated for non-participation (eligible but denied participation), pre-treatment dropout (prior to intervention start), treatment dropout, and loss to follow-up. Descriptive statistics and logistic regression were used in the data analysis.

Results: A majority (80.3 %) were non-participants. Non-participation was predicted by lower level of education, being foreign-born, no experience of counselling for fear of childbirth, multiparity, vaginal delivery (vs. caesarean section and vacuum assisted delivery) and absence of; preeclampsia, anal sphincter injury, and intrapartum foetal distress. Pre-treatment dropout was predicted by absence of severe haemorrhage. Treatment dropout was predicted by vaginal delivery (vs. immediate CS), vertex presentation and good overall birth experience. Loss to follow-up was predicted by vaginal delivery (vs. immediate CS or vacuum-assisted delivery) and absence of intrapartum foetal distress.

For peer review only - http://bmjopen.bmj.com/site/about/guidelines.xhtml

Conclusions: Mothers with no obstetric complications were more likely to not participate and dropout at different time points. Both demographic, antepartum and obstetrical variables are important to attend to while designing procedures to maximize participation in iCBT.

KEYWORDS

Dropout; ICBT; internet-delivered; negative birth experience; non-participation; posttraumatic stress

Strengths and limitations of this study

- A large number of participants from routine health care were included
- Demographic, antepartum, and labour-related/postpartum predictors were investigated at four stages (recruitment, prior to treatment start, during treatment, and at follow-up).
- Neither psychological / psychiatric status or attitudes to internet delivered interventions were investigated in this study but warrants further exploration.

For peer review only

Introduction

The internet has created new opportunities for health care services. Internet-delivered cognitive behavior therapy (iCBT) for various psychological disorders has been developed and investigated in the past decades (1) and the field is growing quickly. The active mechanisms in iCBT are the same as in CBT but differs in the way it is delivered (internet-/computer-based) and increases the availability for evidence based psychological interventions in the society. ICBT is convenient, flexible, and cost-effective for many different psychological disorders (2) it is effective for treatment of depression and several anxiety disorders, and for some diagnoses, iCBT is equally effective as face-to-face CBT (3,4).

Several trials of internet interventions have had problems with high levels of non-adherence, with a majority of the participants never completing treatment (5). Information about dropouts in internet-based interventions is generally poorly reported in the literature (5,6) and one study reported that of 75 reviewed trials, 40% failed to report information about dropouts (7). However, when numbers are reported, they are typically high, especially in self-guided interventions (8) (5). In a review of internet-based treatments, dropout ranged between 2 and 83%, with a weighted average of 31% (9). In a meta-analysis (10), dropout rates of 74% were reported for unguided treatment for depression, whereas the corresponding figure for therapist-supported treatments was 28%. Kuester et al. (11) found an average dropout rate of 23.2% in their meta-analysis of internet-based interventions for PTSD.

The literature is inconsistent regarding the definitions of participants who discontinue before treatment completion (12). Operationalization of adherence varies across trials and limits comparability (13). Eysenbach (14) defines low adherence in internet interventions as "*Nonuse attrition"* (when a participant completes an initial assessment battery but fails to

For peer review only

start the intervention) and "*Dropout attrition"* (when a participant accesses the treatment, but prematurely discontinues it). Other terms, such as "non-compliance," "failure to engage," "premature termination," "attrition," and "dropout" have been used in the literature (12). Melville et al. (9) identified three categories of predictors of dropout: sociodemographic factors and contextual variables, psychological problems, and treatment-related variables – and described that dropout could occur at several different timepoints in iCBT. The following terms for dropout at different timepoints in internet interventions have been suggested: 1. Pre-treatment dropout: when a participant drops out before starting the intervention. 2. Treatment dropout: when a participant drops out after having started the intervention. 3. Follow-up dropout: when a participant completes the intervention but drops out before follow-up measures are completed.

Studies seldom report reasons for non-participation or dropout (15). To better understand who will benefit from internet-based interventions and improve usability and efficacy, there is a need to identify factors related to dropout (16). Adherence to internet-interventions can be influenced by several sociodemographic factors, such as gender, age, and level of education (9,16–19). In a study 96 adult patients with posttraumatic stress reactions were allocated to ten sessions of iCBT or to a waiting list. The dropout rate in the iCBT group was 16%; technical problems and emotional distress due to the treatment interventions were the most frequently reported dropout reasons (20).

The form of an intervention differs in internet treatments, considering amount of material, intensity and support. Some interventions are, e.g., fully therapist-supported with face-to-face sessions or via phone, some offer support via mail, and some do not offer support at all (self-help) (2). Systematic reviews have found that guided internet treatments in general tend to be more effective than non-guided ones (8). Studies seldom report data on the invited persons who decline participation (non-participants). In a randomized controlled trial (RCT)

For peer review only

investigating expressive writing for postpartum physical and psychological health, recruitment was low (10.7% of the invited) (21). The recruited sample derived from a restricted sociodemographic range (high proportion of white Europeans, well-educated, employed, many in professional occupations, older, and more likely to be married).

About 115 000 women give birth in Sweden every year (22). Childbirth is a subjective and multidimensional event that in some cases can lead to a negative childbirth experience. The prevalence of negative childbirth experiences varies (9-45%) in different communities (23–25). For some women (3-4%) the distress of a negative childbirth experience lead to the development of Posttraumatic Stress Disorder Following Childbirth (PTSD FC) (26–31). In Sweden there is no specific treatment recommendation for women with negative birth experiences and/or PTSD FC. So far, only a few randomised controlled trials have investigated the efficacy of different interventions for this population, it is therefore no or little information about how women with negative birth experiences commit and engage in iCBT and similar treatments.

The aim of this study was to investigate a number of possible predictors for non-participation and dropout in an RCT for those with a negative birth experience and/or posttraumatic stress following childbirth (32). The main objective was to investigate demographic, antepartum, and labour-/postpartum related predictors for the following events a) non-participation (eligible women who did not give written consent), b) pre-treatment dropout (i.e., dropout prior to intervention, but after having given informed consent), c) treatment dropout (i.e., dropout during treatment), and d) loss to follow-up (i.e., those who did not complete follow-up measures).

For peer review only

Methods

The STROBE cohort reporting guidelines were used for this publication (33).

Patient and public involvement

Patients or the public were not involved in the design, or conduct, or reporting, or dissemination plans of this study.

Study design

Investigation of single predictors for non-participation, pre-treatment dropout, treatment dropout and loss to follow up, reflecting four consecutive time points (about 8 weeks postpartum, about 10 weeks postpartum, between 10 and 16 weeks postpartum, and after 16 weeks postpartum respectively), for all eligible participants in a longitudinal RCT.

Participants

The current study is a secondary analysis of an RCT for women with negative birth experiences, recruited in routine public health care. Approximately 17,000 women gave birth at *anonymised* Hospital between September 2013 and February 2018, and a majority (n = 1,203) rated their overall birth experience on a Likert scale (0–10), as a standard procedure before discharge. Eligible women (n = 1,523) had a negative birth experience (defined as ≤ 5 on the Likert scale), and/or an immediate caesarean section, and/or a severe postpartum haemorrhage (≥ 2,000 ml). Of 1,523 eligible women, about 20% (n = 300) gave written consent to be part of the RCT (32). The 1,523 eligible women had a mean age of 31.5 years (*SD* = 5.03), participants in the RCT study were 31.7 (4.6) years, and the non-participants age were 31.4 (5.1) years; the majority reported being married or having a partner (84.6 %, n = 1,291), and 50.8% (n = 775) had a university degree. Data on eligible participants are presented in Table 1.

For peer review only

Table 1.

Demographics for the eligible participants (total sample) consisting of those who participated and the non-participants.

	Total<br>*n*=1523<br>*n*(%)	Participants<br>*n*=300<br>*n*(%)	Non-participants<br>*n*=1223<br>*n*(%)
Relationship status			
Married/cohabit	1291 (95.1)	286 (21.1)	1005(74.1)
Single/other	66 (4.9)	8 (0.6)	58(4.3)
Education			
Elementary school	72(5.4)	1 (0.1)	71(5.3)
High school	489(36.6)	82 (6.1)	407(30.5)
University	775(58.0)	209 (54.2)	566(42.4)
Country of birth			
Sweden	953 (76.7)	261 (21)	692 (55.7)
Foreign born	289 (23.3)	25 (2)	264 (21.3)

Note. Missing data; *n*=166 for relationship status, *n*=187 for education, and *n*=281 for country of birth.

Sample size and power

There was no specific sample size calculation for this investigation other than the sample size estimation for the RCT (21) (power was set to 0.8 with a medium effect size) where a total sample size of 130 was needed.

Procedure

For peer review only

Women rated their birth experience as a routine measure at the hospital before discharge. Those with negative birth experiences were contacted via telephone, about eight weeks postpartum. During the telephone calls, the women were informed about the study and those interested in participating were sent study information and a consent form by post. Those who declined at this stage (*n* = 693) were asked about their reason for doing so. In total 530 eligible women did not respond to the invitation, 300 women gave written consent (participants) and 1,223 did not (non-participants). Of the 300 participants, 101 never completed baseline measures (pre-treatment dropouts). The participants who filled out the baseline questionnaires (*n* = 199) were randomized to either treatment as usual (TAU, *n* = 100) or iCBT+TAU (*n* = 99). The iCBT treatment consisted of six treatment modules including psychoeducation and interventions, with therapist support on demand, tailored for women with negative experiences of childbirth (see Appendix 1) (21). Regardless of treatment allocation, local health care providers in accordance with international guidelines treated all participants in the study. TAU included conventional support in accordance with the existing practices at the Department of Obstetrics and Gynecology of the participating hospital. Of the 99 allocated to treatment, a total of 41 were treatment completers (at least three of six steps completed) and 58 were treatment dropouts. All randomized participants (199) were asked to fill out questionnaires six weeks post randomization; 121 completed the follow-up measures and 78 were lost to follow-up, please see figure 1.

Figure 1 about here

Material

Based on previous knowledge about possible causes for non-participation and dropout, predictor variables were categorized into three conceptual categories (demographic, antepartum, and labour-/postpartum related variables). Obstetric data were extracted from

For peer review only

each participant's medical records and questionnaire information was taken from the *anonymised* database. The Care Base Internet Platform, including its web-based part (*anonymised*), was developed within the *anonymised* program. The aim of the *anonymised* research program is to prevent and reduce psychosocial malfunctioning in patients and relatives. The *anonymised* eService is currently being used for interventions and data collection http://www. *anonymised*

Demographic variables

Country of birth (born in *anonymised* /foreign-born), level of education (university/high school/elementary school), relationship status (married/partner or single/other status), and age at delivery (years).

Antepartum variables

Previous caesarean section (no/yes), counselling for fear of childbirth (no/yes), preeclampsia during pregnancy (International Classification of Disease 10th revision (ICD-10 code O14; no/yes), length of pregnancy (number of days based on second trimester ultrasound), and parity (first child/second child/third child or more).

Labour-related/postpartum variables

Mode of delivery (vaginal delivery/emergency caesarean section/immediate caesarean section/vacuum-assisted delivery/elective caesarean section), foetal presentation (vertex presentation/others), manual placenta removal (ICD-10 code O73; no/yes), epidural anaesthesia (ICD-10 code ZXH50; no/yes), intrapartum foetal distress (ICD-10 code O68; no/yes), anal sphincter injury (ICD-10 code O70; no/yes), labour dystocia (ICD-10 code O62; no/yes), severe postpartum haemorrhage (≥ 2,000 ml; no/yes), anaemia (ICD-10 code D59; no/yes), blood transfusion (ICD-10 code Z51.3; no/yes), number of children in the pregnancy (one/two or more), child transferred to neonatal intensive care unit (NICU; no/yes),

For peer review only

breastfeeding problems (ICD-10 code O92; no/yes) and overall birth experience (more severe 0-2 vs. less severe 3-5).

Dependent variables

Non-participation (n = 1,223) vs participation (n=300): eligible women who did not / did return a signed informed consent form.

Pre-treatment dropouts (n = 101) vs pre-treatment completers (n=199): women who gave written consent but did not / did proceed to complete the baseline measurements.

Treatment dropouts (n = 58) vs treatment completers (n=41): women in the iCBT arm who reported activity in 0 to 2 treatment steps of the treatment vs 3 to 6 treatment steps.

Lost to follow-up (n = 78) vs completed follow-up: all randomized women in either treatment arm (iCBT+TAU and TAU) who never completed the post-treatment measures vs those who completed them (n =121).

Statistical analysis

Logistic regression was used to determine predictors of non-participation, pre-treatment dropout, treatment dropout, and loss to follow-up. Odds ratios with 95% confidence intervals and beta values including SE are reported. Missing data were not handled. All available data was used leading to slight differences regarding number of participants in the analyses. Reasons given for non-participation were categorized. SPSS version 26 was used for all analyses.

Ethics

The Regional Ethics Review Board in *anonymised* approved the study (2012/495/1 and amendment 2016/11/16).

Results

Predictors of non-participation

Women with lower levels of education, multiparas and foreign-born were more often non-participants (Table 2). Women who had not been counselled for fear of childbirth, no preeclampsia during pregnancy, no sphincter injury, no intrapartum foetal distress, and those with vaginal delivery were more likely to decline participation, please see Table 2 (and Table A2 for ß,SE).

For peer review only

Table 2.
Odds ratio with 95% CI derived from logistic regression for potential predictors

	Non-participants		Pre-treatment dropout		Treatment dropout		Lost to follow up	
	N	OR 95% CI	N	OR 95% CI	N	OR 95% CI	N	OR 95% CI
Country of birth	1234	3.93(2.58-6.15)***	284	1.09(0.45-2.64)	98	1.43 (0.33-6.06)	198	1.87(0.69-5.08)
Sweden/other	953/281		259/25		89/9		181/17	
Level of education	1336		290		99		199	
University	775	1.0	208	1.0	71	1.0	148	1.0
High school	489	1.83(1.38-2.44)***	81	1.53(0.89-2.62)	27	1.32(0.53-3.28)	50	1.33(0.69-2.55)
Elementary school	72	26.2(3.62-189.9)***	1	na	1	na	1	na
Relationship status	1357		292		97		197	
married-cohabit/other	1291/66	2.06(0.97-4.37)		1.25(0.29-5.35)	95/2	na		na
Age, years	1510	0.99(0.96-1.01)	300	0.97(0.92-1.02)	99	0.95(0.86-1.05)	199	0.95(0.89-1.02)
Previous CS yes/no	1302/189	0.80(0.53-1.19)	31/264	0.78(0.36-1.68)	89/9	1.42(0.33-6.06)	177/19	0.87(0.33-2.32)
Counselling for fear of	1523		300		99		199	
childbirth, no/yes	1376/147	0.50(0.35-0.73)***	254/46	1.06(0.55-2.05)	87/12	0.68(0.18-2.52)	169/30	0.88(0.39-1.97)
Preeclampsia, no/yes	1440/83	0.59(0.36-0.96)*	276/24	1.20(0.51-2.85)	90/9	1.46(0.34-6.22)	184/15	1.39(0.48-4.00)
Pregnancy, days	1507	1.00(0.99-1.00)	299	0.99(0.98-1.01)	99	1.01(0.98-1.04)	198	1.00(0.98-1.02)
Parity	1505		299		99		198	
1st child	838	1	200	1	68	1	134	1
2nd child	448	1.65(1.22-2.22)**	71	0.97(0.55-1.73)	22	1.80(0.65-4.96)	48	1.07(0.54-2.10)
3rd child or more	219	2.15(1.40-3.30)***	28	1.52(0.68-3.40)	9	1.68(0.39-7.26)	16	1.63(0.58-4.60)
Mode of delivery	1523		300		99		199	
Vaginal delivery	783	1	129	1	40	1	82	1
Emergency CS	289	0.71(0.51-1.0)	63	1.0(0.54-1.88)	20	0.33(0.11-1.03)	40	0.74(0.34-1.58)
Immediate CS[1]	186	0.54(0.37-0.79)**	49	0.63(0.30-1.30)	19	0.19(0.06-0.63)**	36	0.42(0.18-0.99)*
Vacuum assisted	198	0.62(0.43-0.91)*	48	0.96(0.48-1.91)	15	0.29(0.84-1.01)	31	0.26(0.10-0.71)**
Elective CS	67	1.01(0.52-1.99)	11	0.17(0.02-1.41)	5	1.33(0.13-13.37)	10	2.57(0.62-10.65)
Foetal presentation	1510		300		99		199	
Vertex / other	1287/223	1.02(0.71-1.46)	256/44	0.53(0.25-1.13)	82/17	0.31(0.11-0.94)*	165/34	1.28(0.61-2.70)
Manual placenta removal	1523		300		99		199	

For peer review only

no / yes	1379/144	1.41(0.88-2.26)	278/22	0.42(0.14-1.26)	93/6	0.33(0.06-1.90)	181/18	0.99(0.36-2.66)
Epidural anaesthesia	1523		300		99		199	
no / yes	813/710	0.86(0.67-1.10)	151/149	1.12(0.69-1.80)	49/50	0.95(0.43-2.12)	102/97	1.52(0.86-2.70)
Intrapartum foetal distress	1523		300		99		199	
no / yes	1234/289	0.67(0.50-0.91)*	228/72	0.76(0.43-1.36)	71/28	0.50(0.21-1.21)	148/51	0.50(0.25-0.99)*
Anal sphincter injury	1523		300		99		199	
no / yes	1447/76	0.48(0.29-0.79)**	275/25	0.92(0.38-2.21)	88/11	1.27(0.35-4.66)	182/17	1.09(0.40-3.00)
Labour dystocia	1523		300		99		199	
no / yes	885/638	0.87(0.67-1.11)	166/134	1.26(0.78-2.04)	55/44	0.74(0.33-1.66)	114/85	0.75(0.42-1.34)
Severe haemmorhage[1]	1523		300		99		199	
no / yes	1329/194	1.19(0.80-1.76)	266/34	0.38(0.15-0.96)*	83/16	0.49(0.17-1.44)	171/28	1.00(0.44-2.28)
Anaemia	1523		300		99		199	
no / yes	1274/249	0.85(0.61-1.18)	246/54	0.57(0.29-1.12)	79/20	0.50(0.19-1.35)	158/41	0.66(0.32-1.38)
Blood transfusion	1523		300		99		199	
no / yes	1340/183	1.01(0.69-1.49)	265/35	0.65(0.29-1.45)	87/12	0.31(0.09-1.09)	173/26	0.53(0.21-1.32)
Children in the pregnancy	1508		299		99		198	
1 child / 2 children	1476/32	1.35(0.52-3.55)	294/5	0.48(0.05-4.40)	97/2	na	194/4	0.51(0.05-4.96)
Child transferred to NICU	1523		300		99		199	
no / yes	1255/268	0.83(0.60-1.14)	241/59	1.21(0.67-2.20)	78/21	0.73(0.28-1.91)	162/37	1.07(0.52-2.22)
Breastfeeding problems	1523		300		99		199	
no / yes	1505/18	0.86(0.28-2.64)	296/54	0.65(0.07-6.36)	98/1	na	196/3	0.77(0.07-8.67)
Overall birth experience[1]	1203		234		72		148	
0-2 / 3-5	305/898	1.02(0.74-1.42)	58/176	0.72(0.38-1.35)	20/52	0.27(0.09-0.79)*	40/108	0.90(0.43-1.88)

[1] inclusion criteria.

* p<.05, **p<.01, ***p<.001

For peer review only

0

Reasons why women declined to take part despite eligibility.

Of the contacted women, 693 actively declined participation and their answers were categorized into different subgroups (Table 3).

Table 3.

Reasons for non-participation (n = 693) given during telephone interview eight weeks postpartum

Reason for non-participation	N	%
Feels fine, does not need any support	326	(47)
Does not speak Swedish	134	(19)
Not interested (no further information)	77	(11)
Feels fine, has already received professional support	35	(5)
Does not feel fine, receiving/waiting for other professional support	35	(5)
Does not have the time	30	(4)
Does not have a computer	16	(2)
Not interested, will not have more kids anyway	14	(2)
Not interested, does not want to think about the delivery	13	(2)
Misunderstood the Likert scale (inclusion), had a positive experience	10	(1.4)
Not comfortable with internet/computer, prefers face-to-face therapy	3	(0.4)

Predictors of pre-treatment dropout

The only significant predictor of pre-treatment dropout was no severe postpartum haemorrhage (i.e., less than 2000 ml; Table 2).

Predictors of treatment dropout

For peer review only

For those randomized to the treatment group, dropout was significantly predicted by mode of delivery, foetal presentation, and overall birth experience (Table 2). Participants with vaginal delivery, vertex presentation and less severe overall birth experience were more likely to drop out from treatment.

Predictors of loss to follow-up

In the analyses of loss to follow-up, absence of intrapartum foetal distress and vaginal delivery (compared with immediate CS and vacuum delivery) predicted loss to follow up (Table 2). An additional analysis showed that being randomised to iCBT+TAU was a significant predictor of loss to follow up OR = 1.84 (95 % CI: 1.04-3.28), B=0.61, SE=0.29, p = .037, where 46 of 99 in iCBT+TAU and 32 of 100 in TAU were lost to follow up.

Discussion

The current study provides an explorative analysis of predictors for non-participation and drop out at different timepoints in an RCT examining iCBT for women with negative birth experiences and/or posttraumatic stress following childbirth (21). Significant predictors for non-participation and dropout were found at different stages in the recruitment process of an RCT. Women with higher education level, without previous children and those born in *anonymised* were more likely to enter into the study. Thereafter, women who had been counselled for fear of birth, experienced complications during the childbirth and with an overall severe birth experience were more likely to stay in the study.

A majority (80.3%) of the eligible women declined participation and our first conclusion was that a large number of those eligible did not see themselves as being in need of iCBT or wanted to take part in a clinical trial. When they were contacted by telephone during the recruitment period, the most frequent reason for declining was “I feel fine/have no

For peer review only

need of any support." Explanations could be that the cut-off for the screening instrument was over-inclusive and/or that the other inclusion criteria (immediate caesarean section and severe postpartum haemorrhage) did not necessarily result in a negative birth experience. The content validity of the one-item Likert scale in the current trial could be discussed, as women may take different aspects of their birth experience into account. Also, the time-point for the rating could be discussed. In the current trial, all women rated their birth experience shortly after giving birth; it is difficult to determine the timepoint that would yield the most accurate rating of the birth experience. However, using a Likert scale as a tool for self-assessment of overall birth experience is well-established in clinical practice and used in research (34,35). A person's perception of their birth experience can change over time and it is important to consider the specific timepoint used in measurement (33). Larsson et al. (36) used a VAS scale (range 1–10) for self-assessment of birth experience at two days, three months, and nine months postpartum. They found that the participants' negative birth experiences decreased over time and suggested that use of a VAS scale was an adequate way to find women in need of follow-up after a negative birth experience.

The analysis included the inclusion criteria (immediate caesarean section, overall birth experience, and severe haemorrhage) as predictors. Non-participation was predicted by vaginal delivery vs. immediate caesarean section. Childbirth without severe haemorrhage predicted pre-treatment dropout. It is known that severe postpartum haemorrhage is a significant risk factor for developing PTSD (37,38). Treatment drop out was predicted by a less severe overall birth experience and vaginal delivery vs. immediate CS. These predictors were inclusion criteria and should be interpreted with caution as mentioned above. The results regarding these predictors must therefore be interpreted with caution. However, the results might be useful for future hypothesis in further research. The three inclusion criteria in this study are experiences that potentially can have serious effects on the mental health of a birth

giving woman. It may be of value to understand more about what type of care (e.g., counselling, therapy), what type of format (e.g., face to face or ICBT) and what level of support (therapist support or pure self-help) is demanded.

Three socio-demographic variables predicted non-participation: lower level of education, multiparity, and being foreign-born. Lower level of education as a greater risk for dropout is consistent with dropout in other iCBT trials (16). Multiparity was also identified as an important predictor of non-participation. The physical and psychological changes of the postpartum period are challenging for first-time mothers and they have lower levels of maternal confidence and higher levels of stress compared with multiparous women (39), which might increase their likelihood of participation. An alternative explanation is that multiparous women have less time to commit to clinical trials compared with first-time mothers. This trial's intervention addressed Swedish-speaking women; foreign-born women might see language as a barrier to participation.

Five antepartum and labour-related/postpartum variables also predicted non-participation. Women without experience of the following were more likely to be non-participants; counselling for fear of childbirth, preeclampsia, anal sphincter injury, intrapartum foetal distress, and vacuum-assisted delivery (vs. vaginal delivery). Women who had been counselled for fear of childbirth had already professionally addressed peripartum psychological problems and might therefore have been more open to support. Preeclampsia, intrapartum foetal distress, and anal sphincter injuries are all severe conditions and motivators for participation. Preeclampsia and severe postpartum haemorrhage are significant threats to the mother and may have devastating or lethal outcomes. Further, both preeclampsia and intrapartum foetal distress are potential threats to the health of the foetus, thus acting as significant stressors for the woman. Instrumental deliveries may be caused by emergency obstetric complications potentially threatening the mother or the child and are very stressful

For peer review only

situations for the woman in their own right (30). The labour related / post-partum predictors show a consistent pattern where women who did not experience these stressful events may not have had enough motivation to seek out help or support.

Pre-treatment dropout was predicted by the absence of severe haemorrhage which is mentioned above. Vertex foetal presentation (vs. other presentation) predicted treatment dropout. This is consistent with the significant predictors for non-participation where those with vertex presentation might not experience this as a stressful event enough to stay in the treatment. It might also be that those with vertex presentation who were randomized to the treatment did not find it helpful or that it did not address their problem fully in order to stay in the treatment.

Predictors for loss to follow-up were vaginal delivery vs. instrumental delivery and absence of intrapartum foetal distress. Occurrence of such events are threats to the foetus which in turn can be very stressful for the mother. Absence of these events might lead to lost interest in devoting time and energy to proceed with the follow-up assessment. Absence of immediate CS was also a significant predictor of loss to follow up and is discussed in relation to the other inclusion criteria/predictors. Finally, randomisation to iCBT+TAU (compared with TAU) was a significant predictor of lost to follow up. A majority of those who were lost to follow up from the iCBT+TAU group were also those who were treatment droputs.

Closer inspection of variables associated with non-participation and dropout can yield insights that can be used in both future research and clinical practice. Knowledge of sub-groups that are more likely to continue and complete study participation provides information about motivational factors and should be applied during the initial recruitment for similar trials. Participants where not asked why they dropped out. We believe that dropout can depend on different factors such as lack of energy and/or interest of being part of a clinical

For peer review only

trial; dropout can also depend on the participant´s experience of not needing the intervention anymore. In this trial women with lover level of education, multipara and foreign born where more often non-participants, perhaps the way of inclusion and the intervention itself must be better adapted to attract those sub-groups in the future. Translation to other languages, using simple language and pictorial material could be ways of improving adherence.

Analyses of predictors of non-participation and dropout are important for evaluating the efficacy of the interventions (6). This explorative study found predictors of non-participation and dropout that should be taken into account in future development of similar interventions. Awareness of characteristics among women who drop out and those who continue, and complete interventions is important and should get more attention during initial recruitment for similar trials.

Strengths

The current study is the first to present data on non-participation and dropouts in iCBT for women with negative birth experiences and/or posttraumatic stress following childbirth. The main strength of this study was the size of the sample and the routine public health care setting as well as consecutive recruitment. All women who gave birth were asked to rate their birth experience on a self-assessed Likert-scale and all women with a low rating were invited. This process increased the likelihood of the results being generalizable to similar clinical contexts. The exploration of dropout predictors from a large cluster of demographic variables and medical/clinical characteristics was another strength. A third strength was that reasons for dropout were explored at different stages in the study process, which allowed analysis of specific timepoints when participants were more likely to end their participation. Analyses of different timepoints for dropout could simplify the analyses of underlying reasons for withdrawal (9).

Limitations

For peer review only

Our study has several limitations that should be noted. Psychological problems and/or treatment-related variables were not available for analyses. Such variables are likely to be strong predictors of non-participation and dropout (9) and should be integrated in future studies. Neither discomfort with the internet or computers were analysed as factors for non-participation or dropout in the current trial. The impact of computer-related factors on adherence has been described previously (17). Further, recruitment to the study was before discharge from the hospital when the experience of birth is fresh. Thus, the eligible sample might have been different if we had asked them at a later time point. However, the assessment of the birth experience was in close conjunction to immediate CS and severe haemorrhage (the two other inclusion criteria).

Conclusions

In this sample, drawn from a large population, predictors were found for non-participation and dropout at different stages in the recruitment process and during the study of an RCT. In summary, both demographic and obstetrical variables are important to attend to for both clinical and research purposes, while designing procedures to maximize participation in iCBT for postpartum women. First time mothers with high level of education and those who had adverse obstetric experiences were more likely to join and stay in the internet intervention.

Conflicts of Interest

All authors of this manuscript declare No known competing interest.

Authorship Contribution statement

All listed authors fulfil ICMJE authorship criteria. Author abbreviations are found in brackets after type of contribution. Writing - original draft (JS), Writing - review & editing (JS, ASS,

For peer review only

FV, ML, ISP, AS, MJ, TP), Conceptualization (ASS, ML, ISP, AS, MJ, TP), Methodology (ASS, ML, ISP, AS, MJ, TP), Formal analysis (JS, ASS, FV, AS, TP), Investigation (JS, ASS, FV, ML, MJ), Data curation (JS, ASS, FV, ML, ISP, AS, MJ, TP), Supervision (ASS, ML, TP), Funding acquisition (ASS, MJ), and Project administration (ASS, ML, ISP, MJ). TP is the guarantor.

Transparency The manuscript's guarantor affirms that the manuscript is an honest, accurate, and transparent account of the study being reported; that no important aspects of the study have been omitted; and that any discrepancies from the study as originally planned have been explained.

Funding. This project was funded by The Regional Research Council (Regionala Forskningsrådet, RFR) grants nr 368901, 308451, and 480141; http://www.researchweb.org/ is/sverige) and Swedish research council funding for clinical research in medicine (ALF) grants nr N/A.

Role of the funding sources The funders of the study had no influence on the study design; in the collection, analysis, and interpretation of data; in the writing of the report; and in the decision to submit the article for publication. All authors confirm the independence from funders and all authors, external and internal, had full access to all of the data (including statistical reports and tables) in the study and take responsibility for the integrity of the data and the accuracy of the data analysis.

Data sharing is available on reasonable request from researchers who provide a methodologically sound proposal. Individual participant data that underlie the results reported in this article, after deidentification will be shared. Proposals should be directed to agneta.skoog_svanberg@kbh.uu.se . To gain access, data requestors will need to sign a data access agreement.

For peer review only

Abbreviations

ICD-10: International Classification of Disease 10th revision

iCBT: Internet-based cognitive behavior therapy

RCT: Randomized controlled trial

TAU: Treatment as usual

For peer review only

References

1. Andersson G. Internet-Delivered Psychological Treatments. Annu Rev Clin Psychol. 2016 Mar 28;12(1):157–79.

2. Spek V, Cuijpers P, NyklíCek I, Riper H, Keyzer J, Pop V. Internet-based cognitive behaviour therapy for symptoms of depression and anxiety: a meta-analysis. Psychol Med. 2007 Mar;37(03):319.

3. Andrews G, Basu A, Cuijpers P, Craske MG, McEvoy P, English CL, et al. Computer therapy for the anxiety and depression disorders is effective, acceptable and practical health care: An updated meta-analysis. J Anxiety Disord. 2018 Apr;55:70–8.

4. Cuijpers P, Donker T, van Straten A, Li J, Andersson G. Is guided self-help as effective as face-to-face psychotherapy for depression and anxiety disorders? A systematic review and meta-analysis of comparative outcome studies. Psychol Med. 2010 Dec;40(12):1943–57.

5. Christensen H, Griffiths KM, Farrer L. Adherence in Internet Interventions for Anxiety and Depression. J Med Internet Res. 2009 Apr 24;11(2):e13.

6. Donkin L, Christensen H, Naismith SL, Neal B, Hickie IB, Glozier N. A Systematic Review of the Impact of Adherence on the Effectiveness of e-Therapies. J Med Internet Res. 2011 Aug 5;13(3):e52.

7. Kiluk BD, Sugarman DE, Nich C, Gibbons CJ, Martino S, Rounsaville BJ, et al. A Methodological Analysis of Randomized Clinical Trials of Computer-Assisted Therapies for Psychiatric Disorders: Toward Improved Standards for an Emerging Field. Am J Psychiatry. 2011 Aug;168(8):790–9.

8. Baumeister H, Reichler L, Munzinger M, Lin J. The impact of guidance on Internet-based mental health interventions — A systematic review. Internet Interv. 2014 Oct;1(4):205–15.

9. Melville KM, Casey LM, Kavanagh DJ. Dropout from Internet-based treatment for psychological disorders. Br J Clin Psychol. 2010 Nov;49(4):455–71.

10. Richards D, Richardson T. Computer-based psychological treatments for depression: A systematic review and meta-analysis. Clin Psychol Rev. 2012 Jun;32(4):329–42.

11. Kuester A, Niemeyer H, Knaevelsrud C. Internet-based interventions for posttraumatic stress: A meta-analysis of randomized controlled trials. Clin Psychol Rev. 2016 Feb;43:1–16.

12. Melville KM, Casey LM, Kavanagh DJ. Psychological treatment dropout among pathological gamblers. Clin Psychol Rev. 2007 Dec;27(8):944–58.

For peer review only

13. Sieverink F, Kelders SM, van Gemert-Pijnen JE. Clarifying the Concept of Adherence to eHealth Technology: Systematic Review on When Usage Becomes Adherence. J Med Internet Res. 2017 Dec 6;19(12):e402.
14. Eysenbach G. The Law of Attrition. J Med Internet Res. 2005 Mar 31;7(1):e11.
15. Gul RB, Ali PA. Clinical trials: the challenge of recruitment and retention of participants. J Clin Nurs. 2010 Jan;19(1–2):227–33.
16. Karyotaki E, Kleiboer A, Smit F, Turner DT, Pastor AM, Andersson G, et al. Predictors of treatment dropout in self-guided web-based interventions for depression: an 'individual patient data' meta-analysis. Psychol Med. 2015 Oct;45(13):2717–26.
17. Beatty L, Binnion C. A Systematic Review of Predictors of, and Reasons for, Adherence to Online Psychological Interventions. Int J Behav Med. 2016 Dec;23(6):776–94.
18. Edmonds M, Hadjistavropoulos HD, Schneider LH, Dear BF, Titov N. Who benefits most from therapist-assisted internet-delivered cognitive behaviour therapy in clinical practice? Predictors of symptom change and dropout. J Anxiety Disord. 2018 Mar;54:24–32.
19. Hedman E, Ljótsson B, Kaldo V, Hesser H, El Alaoui S, Kraepelien M, et al. Effectiveness of Internet-based cognitive behaviour therapy for depression in routine psychiatric care. J Affect Disord. 2014 Feb;155:49–58.
20. Knaevelsrud C, Maercker A. Internet-based treatment for PTSD reduces distress and facilitates the development of a strong therapeutic alliance: a randomized controlled clinical trial. BMC Psychiatry. 2007;7(1):13.
21. Crawley R, Ayers S, Button S, Thornton A, Field AP, Lee S, et al. Feasibility and acceptability of expressive writing with postpartum women: a randomised controlled trial. BMC Pregnancy Childbirth [Internet]. 2018 Dec;18(1).
22. Socialstyrelsen. Statistik om graviditeter, förlossningar och nyfödda barn 2019 [Internet]. Stockholm; 2020 [cited 2021 Sep 8] p. 4. Report No.: Art.nr: 2020-12-7051. Available from: https://www.socialstyrelsen.se/globalassets/sharepoint-dokument/artikelkatalog/statistik/2020-12-7051.pdf
23. de Graaff LF, Honig A, van Pampus MG, Stramrood CAI. Preventing post-traumatic stress disorder following childbirth and traumatic birth experiences: a systematic review. Acta Obstet Gynecol Scand. 2018 Jun;97(6):648–56.
24. Haagen JFG, Moerbeek M, Olde E, van der Hart O, Kleber RJ. PTSD after childbirth: A predictive ethological model for symptom development. J Affect Disord. 2015 Oct;185:135–43.

For peer review only

25. Alcorn KL, O'Donovan A, Patrick JC, Creedy D, Devilly GJ. A prospective longitudinal study of the prevalence of post-traumatic stress disorder resulting from childbirth events. Psychol Med. 2010 Nov;40(11):1849–59.
26. Wijma K, Söderquist J, Wijma B. Posttraumatic stress disorder after childbirth: A cross sectional study. J Anxiety Disord. 1997 Nov;11(6):587–97.
27. Olde E, van der Hart O, Kleber RJ, van Son MJM, Wijnen HAA, Pop VJM. Peritraumatic Dissociation and Emotions as Predictors of PTSD Symptoms Following Childbirth. J Trauma Dissociation. 2005 Sep 13;6(3):125–42.
28. Garthus-Niegel S, von Soest T, Vollrath ME, Eberhard-Gran M. The impact of subjective birth experiences on post-traumatic stress symptoms: a longitudinal study. Arch Womens Ment Health. 2013 Feb;16(1):1–10.
29. Dekel S, Stuebe C, Dishy G. Childbirth Induced Posttraumatic Stress Syndrome: A Systematic Review of Prevalence and Risk Factors. Front Psychol. 2017 Apr 11;8.
30. Grekin R, O'Hara MW. Prevalence and risk factors of postpartum posttraumatic stress disorder: A meta-analysis. Clin Psychol Rev. 2014 Jul;34(5):389–401.
31. Yildiz PD, Ayers S, Phillips L. The prevalence of posttraumatic stress disorder in pregnancy and after birth: A systematic review and meta-analysis. J Affect Disord. 2017 Jan;208:634–45.
32. Sjömark J, Parling T, Jonsson M, Larsson M, Skoog Svanberg A. A longitudinal, multi-centre, superiority, randomized controlled trial of internet-based cognitive behavioural therapy (iCBT) versus treatment-as-usual (TAU) for negative experiences and posttraumatic stress following childbirth: the JUNO study protocol. BMC Pregnancy Childbirth. 2018;18(387).
33. Elm E von, Altman D, Egger M, Pocock S, Gotzsche P, Vandenbroucke J. The Strengthening the Reporting of Observational Studies in Epidemiology (STROBE) Statement: guidelines for reporting observational studies. https://www.equator-network.org/reporting-guidelines/strobe/
34. Waldenström U. Why do some women change their opinion about childbirth over time? Birth. 2004;31(2):102–7.
35. Waldenström U, Hildingsson I, Rubertsson C, Raadestad I. A negative birth experience: prevalence and risk factors in a national sample. Birth. 2004;31(1):17–27.
36. Larsson C, Saltvedt S, Edman G, Wiklund I, Andolf E. Factors independently related to a negative birth experience in first-time mothers. Sex Reprod Healthc. 2011 Apr;2(2):83–9.
37. van Steijn ME, Scheepstra KWF, Zaat TR, van Rooijen DE, Stramrood CAI, Dijksman

For peer review only

LM, et al. Severe postpartum hemorrhage increases risk of posttraumatic stress disorder: a prospective cohort study. J Psychosom Obstet Gynecol. 2020 Mar 17;1–11.

38. Michelet D, Ricbourg A, Gosme C, Rossignol M, Schurando P, Barranger E, et al. Emergency hysterectomy for life-threatening postpartum haemorrhage: Risk factors and psychological impact. Gynécologie Obstétrique Fertil. 2015 Dec;43(12):773–9.

39. Kristensen IH, Simonsen M, Trillingsgaard T, Pontoppidan M, Kronborg H. First-time mothers' confidence mood and stress in the first months postpartum. A cohort study. Sex Reprod Healthc. 2018 Oct;17:43–9.

Figure caption.

Figure 1. Flowchart

For peer review only

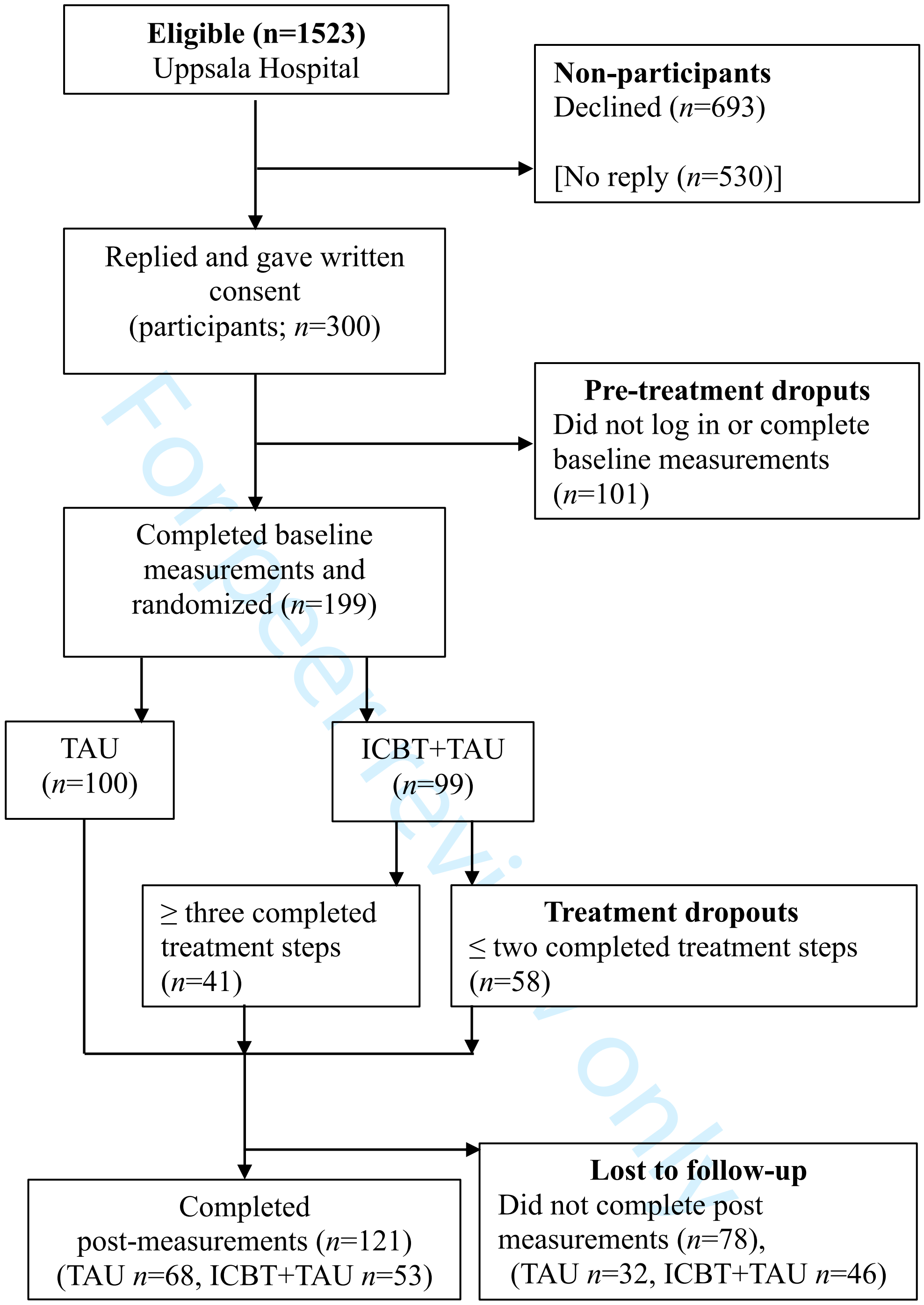

Figure 1. Flowchart

For peer review only

Appendix 1 Week by week content of the iCBT treatment

Week	Content
1st	Information, psychoeducation, breathing retraining
2nd	Vignettes, common symptoms, fear and avoidance
3rd	Depressive symptoms, significance of relations,“reflective listening”
4th	Exposure, talking about the childbirth
5th	Managing anxiety and depressive symptoms, psychological health, values, recovery
6th	Summary, repetition and relapse prevention

Note. Every week contains homework assignments based on the content of the module

For peer review only

Table 2.
Odds ratio with 95% CI, Beta values, and SE derived from logistic regression, for potential predictors

	Non-participants		Pre-treatment dropout		Treatment dropout		Lost to follow up	
	N	OR 95% CI<br>*B*, S.E	N	OR 95% CI<br>*B*, S.E	N	OR 95% CI<br>*B*, S.E	N	OR 95% CI<br>*B*, S.E
Country of birth<br>Sweden/other	1234<br>953/281	3.93(2.58-6.15)***<br>1.38, 0.22	284<br>259/25	1.09(0.45-2.64)<br>0.09, 0.45	98<br>89/9	1.43 (0.33-6.06)<br>0.35, 0.74	198<br>181/17	1.87(0.69-5.08)<br>0.63, 0.51
Level of education	1336		290		99		199	
University	775	1.0	208	1.0	71	1.0	148	1.0
High school	489	1.83(1.38-2.44)***<br>0.61, 0.15	81	1.53(0.89-2.62)<br>0.43, 0.28	27	1.32(0.53-3.28)<br>0.28, 0.46	50	1.33(0.69-2.55)<br>0.28, 0.33
Elementary school	72	26.2(3.62-189.9)***<br>3.27, 1.01	1	na	1	na	1	na
Relationship status<br>married-cohabit/other	1357<br>1291/66	2.06(0.97-4.37)<br>0.72, 0.38	292	1.25(0.29-5.35)<br>0.256, 0.74	97<br>95/2	na	197<br>192/5	na
Age<br>years	1510	0.99(0.96-1.01)<br>-0.12, 0.013	300	0.97(0.92-1.02)<br>-0.034, 0.86	99	0.95(0.86-1.05)<br>-0.52, 0.05	199	0.95(0.89-1.02)<br>-0.048, 0.03
Previous CS<br>no / yes	1491<br>1302/189	0.80(0.53-1.19)<br>-0.23, 0.21	295<br>31/264	0.78(0.36-1.68)<br>-0.25, 0.39	98<br>89/9	1.42(0.33-6.06)<br>0.35, 0.74	196<br>177/19	0.87(0.33-2.32)<br>-0.14, 0.50
Counselling for fear of childbirth, no / yes	1523<br>1376/147	0.50(0.35-0.73)***<br>-0.69, 0.19	300<br>254/46	1.06(0.55-2.05)<br>0.06, 034	99<br>87/12	0.68(0.18-2.52)<br>-0.40, 0.62	199<br>169/30	0.88(0.39-1.97)<br>-0.13, 0.41
Preeclampsia<br>no / yes	1523<br>1440/83	0.59(0.36-0.96)*<br>-0.53, 0.25	300<br>276/24	1.20(0.51-2.85)<br>0.18, 0.44	99<br>90/9	1.46(0.34-6.22)<br>0.38, 0.74	199<br>184/15	1.39(0.48-4.00)<br>0.33, 0.54
Length of pregnancy<br>days	1507	1.00(0.99-1.00)<br>-0.003, 0.004	299	0.99(0.98-1.01)<br>-0.008, 0.008	99	1.01(0.98-1.04)<br>0.01, 0.013	198	1.00(0.98-1.02)<br>0.002, 0.01
Parity	1505		299		99		198	
1st child	838	1	200	1	68	1	134	1

For peer review only

2nd child	448	1.65(1.22-2.22)**<br>0.50, 0.15	71	0.97(0.55-1.73)<br>-0.28, 029	22	1.80(0.65-4.96)<br>0.58, 0.52	48	1.07(0.54-2.10)<br>0.06, 0.34
3rd child or more	219	2.15(1.40-3.30)***<br>0.77, 0.081	28	1.52(0.68-3.40)<br>0.42, 0.41	9	1.68(0.39-7.26)<br>0.52, 0.75	16	1.63(0.58-4.60)<br>0.49, 0.53
Mode of delivery	1523		300		99		199	
Vaginal delivery	783	1	129	1	40	1	82	1
Emergency CS	289	0.71(0.51-1.0)<br>-0.34, 0.17	63	1.0(0.54-1.88)<br>0.003, 0.32	20	0.33(0.11-1.03)<br>-1.1, 0.58	40	0.74(0.34-1.58)<br>-0.31, 0.39
Immediate CS[1]	186	0.54(0.37-0.79)**<br>-0.61, 0.19	49	0.63(0.30-1.30)<br>-0.46, 0.37	19	0.19(0.06-0.63)**<br>-1.64, 0.60	36	0.42(0.18-0.99)*<br>-0.86, 0.43
Vacuum assisted	198	0.62(0.43-0.91)*<br>-0.475, 0.19	48	0.96(0.48-1.91)<br>-0.04, 0.35	15	0.29(0.84-1.01)<br>-1.23, 0.63	31	0.26(0.10-0.71)**<br>-1.33, 0.51
Elective CS	67	1.01(0.52-1.99)<br>0.01, 0.34	11	0.17(0.02-1.41)<br>-1.75, 1.06	5	1.33(0.13-13.37)<br>0.29, 1.18	10	2.57(0.62-10.65)<br>0.95, 0.73
Foetal presentation	1510	1.02(0.71-1.46)	300	0.53(0.25-1.13)	99	0.31(0.11-0.94)*	199	1.28(0.61-2.70)
Vertex / other	1287/223	0.20, 0.18	256/44	-0.63, 0.38	82/17	-1.16, 0.56	165/34	0.24, 0.38
Manual placenta removal	1523	1.41(0.88-2.26)	300	0.42(0.14-1.26)	99	0.33(0.06-1.90)	199	0.99(0.36-2.66)
no / yes	1379/144	0.346, 0.24	278/22	-0.88, 0.57	93/6	-1.11, 0.89	181/18	-0.014, 0.51
Epidural anaesthesia	1523	0.86(0.67-1.10)	300	1.12(0.69-1.80)	99	0.95(0.43-2.12)	199	1.52(0.86-2.70)
no / yes	813/710	-0.15, 0.13	151/149	0.11, 024	49/50	-0.049, 0.41	102/97	0.42, 0.29
Intrapartum foetal distress	1523	0.67(0.50-0.91)*	300	0.76(0.43-1.36)	99	0.50(0.21-1.21)	199	0.50(0.25-0.99)*
no / yes	1234/289	-0.39, 0.15	228/72	-0.27, 0.29	71/28	-0.69, 0.45	148/51	-0.70, 0.36
Anal sphincter injury	1523	0.48(0.29-0.79)**	300	0.92(0.38-2.21)	99	1.27(0.35-4.66)	199	1.09(0.40-3.00)
no / yes	1447/76	-0.73, 0.25	275/25	-0.82, 045	88/11	0.24, 0.66	182/17	0.09, 0.52
Labour dystocia	1523	0.87(0.67-1.11)	300	1.26(0.78-2.04)	99	0.74(0.33-1.66)	199	0.75(0.42-1.34)
no / yes	885/638	-0.14, 0.13	166/134	0.23, 0.25	55/44	-0.3, 0.41	114/85	-0.29, 0.30
Severe haemmorhage[1]	1523	1.19(0.80-1.76)	300	0.38(0.15-0.96)*	99	0.49(0.17-1.44)	199	1.00(0.44-2.28)

For peer review only

no / yes	1329/194	0.17, 0.20	266/34	-0.95, 0.47	83/16	-0.72, 0.55	171/28	0.004, 0.42
Anaemia	1523	0.85(0.61-1.18)	300	0.57(0.29-1.12)	99	0.50(0.19-1.35)	199	0.66(0.32-1.38)
no / yes	1274/249	-0.16, 0.17	246/54	-0.56, 0.35	79/20	-0.69, 0.51	158/41	-0.41, 0.37
Blood transfusion	1523	1.01(0.69-1.49)	300	0.65(0.29-1.45)	99	0.31(0.09-1.09)	199	0.53(0.21-1.32)
no / yes	1340/183	0.011, 0.20	265/35	-0.43, 0.41	87/12	-1.19, 0.65	173/26	-0.64, 0.47
Children in the pregnancy	1508	1.35(0.52-3.55)	299	0.48(0.05-4.40)	99	na	198	0.51(0.05-4.96)
1 child / 2 children	1476/32	0.30, 0.49	294/5	-0.72, 1.12	97/2		194/4	-0.68, 1.16
Child transferred to NICU	1523	0.83(0.60-1.14)	300	1.21(0.67-2.20)	99	0.73(0.28-1.91)	199	1.07(0.52-2.22)
no / yes	1255/268	-0.19, 0.16	241/59	0.20, 0.30	78/21	-0.32, 0.49	162/37	0.069, 0.37
Breastfeeding problems	1523	0.86(0.28-2.64)	300	0.65(0.07-6.36)	99	na	199	0.77(0.07-8.67)
no / yes	1505/18	-0.15, 0.57	296/54	-0.43, 1.16	98/1		196/3	-0.26, 1.23
Overall birth experience[1]	1203	1.02(0.74-1.42)	234	0.72(0.38-1.35)	72	0.27(0.09-0.79)*	148	0.90(0.43-1.88)
0-2 / 3-5	305/898	0.023, 0.17	58/176	-0.34, 0.32	20/52	-1.31, 0.55	40/108	-0.11, 0.38

[1] inclusion criteria.

* p<.05, **p<.01, ***p<.001

For peer review only

Reporting checklist for cohort study.

Based on the STROBE cohort guidelines.

Instructions to authors

Complete this checklist by entering the page numbers from your manuscript where readers will find each of the items listed below.

Your article may not currently address all the items on the checklist. Please modify your text to include the missing information. If you are certain that an item does not apply, please write "n/a" and provide a short explanation.

Upload your completed checklist as an extra file when you submit to a journal.

In your methods section, say that you used the STROBE cohortreporting guidelines, and cite them as:

von Elm E, Altman DG, Egger M, Pocock SJ, Gotzsche PC, Vandenbroucke JP. The Strengthening the Reporting of Observational Studies in Epidemiology (STROBE) Statement: guidelines for reporting observational studies.

		Reporting Item	Page Number
**Title and abstract**		Internet-based Cognitive Behavior Therapy (iCBT) for women with negative birth experiences and/or posttraumatic stress following childbirth: Prevalence and predictors of non-participation and dropout	
Title	#1a	Indicate the study's design with a commonly used term in the title or the abstract	Title page
Abstract	#1b	Provide in the abstract an informative and balanced summary of what was done and what was found	Abstract page
**Introduction**			
Background / rationale	#2	Explain the scientific background and rationale for the investigation being reported	5-7
Objectives	#3	State specific objectives, including any prespecified hypotheses	7
**Methods**			
Study design	#4	Present key elements of study design early in the paper	8

For peer review only

Setting	#5	Describe the setting, locations, and relevant dates, including periods of recruitment, exposure, follow-up, and data collection	8
Eligibility criteria	#6a	Give the eligibility criteria, and the sources and methods of selection of participants. Describe methods of follow-up.	8-10
Eligibility criteria	#6b	For matched studies, give matching criteria and number of exposed and unexposed	n.a
Variables	#7	Clearly define all outcomes, exposures, predictors, potential confounders, and effect modifiers. Give diagnostic criteria, if applicable	10-12
Data sources / measurement	#8	For each variable of interest give sources of data and details of methods of assessment (measurement). Describe comparability of assessment methods if there is more than one group. Give information separately for for exposed and unexposed groups if applicable.	10-12
Bias	#9	Describe any efforts to address potential sources of bias	18-23
Study size	#10	Explain how the study size was arrived at	9
Quantitative variables	#11	Explain how quantitative variables were handled in the analyses. If applicable, describe which groupings were chosen, and why	10-12
Statistical methods	#12a	Describe all statistical methods, including those used to control for confounding	12
Statistical methods	#12b	Describe any methods used to examine subgroups and interactions	n.a.
Statistical methods	#12c	Explain how missing data were addressed	12
Statistical methods	#12d	If applicable, explain how loss to follow-up was addressed	12
Statistical methods	#12e	Describe any sensitivity analyses	n.a
**Results**			
Participants	#13a	Report numbers of individuals at each stage of study—eg numbers potentially eligible, examined for eligibility, confirmed eligible, included in the study, completing follow-up, and analysed. Give	fig 1, table 2

For peer review only

		information separately for for exposed and unexposed groups if applicable.	
Participants	#13b	Give reasons for non-participation at each stage	Fig 1
Participants	#13c	Consider use of a flow diagram	Fig 1
Descriptive data	#14a	Give characteristics of study participants (eg demographic, clinical, social) and information on exposures and potential confounders. Give information separately for exposed and unexposed groups if applicable.	Table 1
Descriptive data	#14b	Indicate number of participants with missing data for each variable of interest	Table 2
Descriptive data	#14c	Summarise follow-up time (eg, average and total amount)	10
Outcome data	#15	Report numbers of outcome events or summary measures over time. Give information separately for exposed and unexposed groups if applicable.	Table 2
Main results	#16a	Give unadjusted estimates and, if applicable, confounder-adjusted estimates and their precision (eg, 95% confidence interval). Make clear which confounders were adjusted for and why they were included	Table 2
Main results	#16b	Report category boundaries when continuous variables were categorized	Table 2
Main results	#16c	If relevant, consider translating estimates of relative risk into absolute risk for a meaningful time period	n.a
Other analyses	#17	Report other analyses done—eg analyses of subgroups and interactions, and sensitivity analyses	n.a
**Discussion**			

For peer review only

Key results	#18	Summarise key results with reference to study objectives	18-22
Limitations	#19	Discuss limitations of the study, taking into account sources of potential bias or imprecision. Discuss both direction and magnitude of any potential bias.	22-23
Interpretation	#20	Give a cautious overall interpretation considering objectives, limitations, multiplicity of analyses, results from similar studies, and other relevant evidence.	18-23
Generalisability	#21	Discuss the generalisability (external validity) of the study results	18-23
**Other Information**			
Funding	#22	Give the source of funding and the role of the funders for the present study and, if applicable, for the original study on which the present article is based	24

None The STROBE checklist is distributed under the terms of the Creative Commons Attribution License CC-BY. This checklist can be completed online using https://www.goodreports.org/, a tool made by the EQUATOR Network in collaboration with Penelope.ai

For peer review only

BMJ Open

Antepartum and labour related single predictors of non-participation, dropout, and lost to follow up in a randomised controlled trial comparing internet-based cognitive behaviour therapy with treatment as usual for women with negative birth experiences and/or post-traumatic stress following childbirth.

Journal:	*BMJ Open*
Manuscript ID	bmjopen-2022-063214.R1
Article Type:	Original research
Date Submitted by the Author:	24-Aug-2022
Complete List of Authors:	Sjömark, Josefin; Uppsala Universitet, Department of Women’s and Children’s Health<br>Svanberg, Agneta ; Uppsala Universitet, Department of Women's and Children's Health<br>Viirman, Frida; Uppsala Universitet, Department of Women’s and Children’s Health<br>Larsson, Margareta; Uppsala University, Dep of Women's and Children's Health<br>Poromaa, Inger; Uppsala Universitet, Department of Women’s and Children’s Health<br>Skalkidou, Alkistis; Uppsala University, Department of Women’s and Children’s Health<br>Jonsson, Maria; Uppsala University, Department of Women’s and Children’s Health<br>Parling, Thomas; Uppsala University, Department of Women’s and Children’s Health; Karolinska Institutet & Stockholm Health Care Services Region Stockholm, clinical neuroscience
**Primary Subject Heading**:	Mental health
Secondary Subject Heading:	Obstetrics and gynaecology
Keywords:	GYNAECOLOGY, MENTAL HEALTH, OBSTETRICS, Adult psychiatry < PSYCHIATRY, Anxiety disorders < PSYCHIATRY

SCHOLARONE™
Manuscripts

I, the Submitting Author has the right to grant and does grant on behalf of all authors of the Work (as defined in the below author licence), an exclusive licence and/or a non-exclusive licence for contributions from authors who are: i) UK Crown employees; ii) where BMJ has agreed a CC-BY licence shall apply, and/or iii) in accordance with the terms applicable for US Federal Government officers or employees acting as part of their official duties; on a worldwide, perpetual, irrevocable, royalty-free basis to BMJ Publishing Group Ltd ("BMJ") its licensees and where the relevant Journal is co-owned by BMJ to the co-owners of the Journal, to publish the Work in this journal and any other BMJ products and to exploit all rights, as set out in our licence.

The Submitting Author accepts and understands that any supply made under these terms is made by BMJ to the Submitting Author unless you are acting as an employee on behalf of your employer or a postgraduate student of an affiliated institution which is paying any applicable article publishing charge ("APC") for Open Access articles. Where the Submitting Author wishes to make the Work available on an Open Access basis (and intends to pay the relevant APC), the terms of reuse of such Open Access shall be governed by a Creative Commons licence – details of these licences and which Creative Commons licence will apply to this Work are set out in our licence referred to above.

Other than as permitted in any relevant BMJ Author's Self Archiving Policies, I confirm this Work has not been accepted for publication elsewhere, is not being considered for publication elsewhere and does not duplicate material already published. I confirm all authors consent to publication of this Work and authorise the granting of this licence.

For peer review only

Antepartum and labour related single predictors of non-participation, dropout, and lost to follow up in a randomised controlled trial comparing internet-based cognitive behaviour therapy with treatment as usual for women with negative birth experiences and/or post-traumatic stress following childbirth.

Josefin Sjömark[1] MS; Agneta Skoog Svanberg[1] professor; Frida Viirman[1] BSc; Margareta Larsson[1] associate professor; Inger Sundström Poromaa[1] professor; Alkistis Skalkidou[1] professor; Maria Jonsson[1] associate professor; Thomas Parling[1,2] PhD

[1] Department of Women's and Children's Health, Uppsala University, SE-751 85 Uppsala, Sweden. | josefin.sjomark@kbh.uu.se ; agneta.skoog_svanberg@kbh.uu.se ; frida.viirman@kbh.uu.se ; Margareta.Larsson@kbh.uu.se ; Inger.Sundstrom@kbh.uu.se ; alkistis.skalkidou@kbh.uu.se ; maria.jonsson@kbh.uu.se ; Thomas.parling@kbh.uu.se

[2] Centre for Psychiatry Research, Department of Clinical Neuroscience, Karolinska Institutet, & Stockholm Health Care Services, Region Stockholm | thomas.parling@ki.se

Trial Registration: This study is a dropout investigation of a randomized controlled trial (RCT). The RCT trial registration: ISRCTN39318241. Date for registration 12/01/2017, retrospectively registered.

Word count: 3007

Acknowledgements

We would like to thank statistician Per Wikman for help with structuring the database.

Corresponding Author:

For peer review only

Thomas Parling

Department of Women's and Children's Health

Uppsala University

Dag Hammarskjölds väg 14B

SE-751 85 Uppsala

Sweden

Phone: 46 70 2058007

Email: thomas.parling@kbh.uu.se

For peer review only

Abstract

Objectives: Internet-based interventions are often hampered by high dropout rates. The number of individuals who decline to participate or drop out are reported, but reasons for dropout are not. Identification of barriers to participation and predictors of dropout may help improve the efficacy of internet-based clinical trials. The aim was to investigate a large number of possible predictors for non-participation and dropout in a randomized controlled trial for women with a negative birth experience and/or post-traumatic stress following childbirth.

Setting: A childbirth clinic at a university hospital in Sweden.

Participants: The sample included 1,523 women who gave birth between September 2013 and February 2018. All women who rated an overall negative birth experience on a Likert scale, and/or had an immediate caesarean section (CS), and/or severe postpartum haemorrhage (≥ 2,000 ml) were eligible.

Methods: Demographic, antepartum, and labour-related/postpartum predictors were investigated for non-participation (eligible but denied participation), pre-treatment dropout (prior to intervention start), treatment dropout, and loss to follow-up. Descriptive statistics and logistic regression were used in the data analysis.

Results: A majority (80.3 %) were non-participants. Non-participation was predicted by lower level of education, being foreign-born, no experience of counselling for fear of childbirth, multiparity, vaginal delivery (vs. caesarean section and vacuum assisted delivery) and absence of; preeclampsia, anal sphincter injury, and intrapartum foetal distress. Pre-treatment dropout was predicted by absence of severe haemorrhage. Treatment dropout was predicted by vaginal delivery (vs. immediate CS), vertex presentation and good overall birth experience. Loss to follow-up was predicted by vaginal delivery (vs. immediate CS or vacuum-assisted delivery) and absence of intrapartum foetal distress.

For peer review only

Conclusions: Mothers with no obstetric complications were more likely to not participate and dropout at different time points. Both demographic, antepartum and obstetrical variables are important to attend to while designing procedures to maximize participation in iCBT.

KEYWORDS

Dropout; ICBT; internet-delivered; negative birth experience; non-participation; posttraumatic stress

Strengths and limitations of this study

- A large number of participants from routine health care were included
- Demographic, antepartum, and labour-related/postpartum predictors were investigated at four stages (recruitment, prior to treatment start, during treatment, and at follow-up).
- Neither psychological / psychiatric status or attitudes to internet delivered interventions were investigated in this study but warrants further exploration.

For peer review only

Introduction

The internet has created new opportunities for health care services. Internet-delivered cognitive behavior therapy (iCBT) for various psychological disorders has been developed and investigated in the past decades (1) and the field is growing quickly. The active mechanisms in iCBT are the same as in CBT but differs in the way it is delivered (internet-/computer-based) and increases the availability for evidence based psychological interventions in the society. ICBT is convenient, flexible, and cost-effective for many different psychological disorders (2) it is effective for treatment of depression and several anxiety disorders, and for some diagnoses, iCBT is equally effective as face-to-face CBT (3,4).

Several trials of internet interventions have had problems with high levels of non-adherence, with a majority of the participants never completing treatment (5). Information about dropouts in internet-based interventions is generally poorly reported in the literature (5,6) and one study reported that of 75 reviewed trials, 40% failed to report information about dropouts (7). However, when numbers are reported, they are typically high, especially in self-guided interventions (8) (5). In a review of internet-based treatments, dropout ranged between 2 and 83%, with a weighted average of 31% (9). In a meta-analysis (10), dropout rates of 74% were reported for unguided treatment for depression, whereas the corresponding figure for therapist-supported treatments was 28%. Kuester et al. (11) found an average dropout rate of 23.2% in their meta-analysis of internet-based interventions for PTSD.

The literature is inconsistent regarding the definitions of participants who discontinue before treatment completion (12). Operationalization of adherence varies across trials and limits comparability (13). Eysenbach (14) defines low adherence in internet interventions as "*Nonuse attrition"* (when a participant completes an initial assessment battery but fails to

For peer review only

start the intervention) and "*Dropout attrition"* (when a participant accesses the treatment, but prematurely discontinues it). Other terms, such as "non-compliance," "failure to engage," "premature termination," "attrition," and "dropout" have been used in the literature (12). Melville et al. (9) identified three categories of predictors of dropout: sociodemographic factors and contextual variables, psychological problems, and treatment-related variables – and described that dropout could occur at several different timepoints in iCBT. The following terms for dropout at different timepoints in internet interventions have been suggested: 1. Pre-treatment dropout: when a participant drops out before starting the intervention. 2. Treatment dropout: when a participant drops out after having started the intervention. 3. Follow-up dropout: when a participant completes the intervention but drops out before follow-up measures are completed.

Studies seldom report reasons for non-participation or dropout (15). To better understand who will benefit from internet-based interventions and improve usability and efficacy, there is a need to identify factors related to dropout (16). Adherence to internet-interventions can be influenced by several sociodemographic factors, such as gender, age, and level of education (9,16–19). In a study 96 adult patients with posttraumatic stress reactions were allocated to ten sessions of iCBT or to a waiting list. The dropout rate in the iCBT group was 16%; technical problems and emotional distress due to the treatment interventions were the most frequently reported dropout reasons (20).

The form of an intervention differs in internet treatments, considering amount of material, intensity and support. Some interventions are, e.g., fully therapist-supported with face-to-face sessions or via phone, some offer support via mail, and some do not offer support at all (self-help) (2). Systematic reviews have found that guided internet treatments in general tend to be more effective than non-guided ones (8). Studies seldom report data on the invited persons who decline participation (non-participants). In a randomized controlled trial (RCT)

For peer review only

investigating expressive writing for postpartum physical and psychological health, recruitment was low (10.7% of the invited) (21). The recruited sample derived from a restricted sociodemographic range (high proportion of white Europeans, well-educated, employed, many in professional occupations, older, and more likely to be married).

About 115 000 women give birth in Sweden every year (22). Childbirth is a subjective and multidimensional event that in some cases can lead to a negative childbirth experience. The prevalence of negative childbirth experiences varies (9-45%) in different communities (23–25). For some women (3-4%) the distress of a negative childbirth experience lead to the development of Posttraumatic Stress Disorder Following Childbirth (PTSD FC) (26–31). In Sweden there is no specific treatment recommendation for women with negative birth experiences and/or PTSD FC. So far, only a few randomised controlled trials have investigated the efficacy of different interventions for this population, it is therefore no or little information about how women with negative birth experiences commit and engage in iCBT and similar treatments.

The aim of this study was to investigate a number of possible predictors for non-participation and dropout in an RCT for those with a negative birth experience and/or posttraumatic stress following childbirth (32). The main objective was to investigate demographic, antepartum, and labour-/postpartum related predictors for the following events a) non-participation (eligible women who did not give written consent), b) pre-treatment dropout (i.e., dropout prior to intervention, but after having given informed consent), c) treatment dropout (i.e., dropout during treatment), and d) loss to follow-up (i.e., those who did not complete follow-up measures).

For peer review only

Methods

The STROBE cohort reporting guidelines were used for this publication (33).

Patient and public involvement

Patients or the public were not involved in the design, or conduct, or reporting, or dissemination plans of this study.

Study design

Investigation of single predictors for non-participation, pre-treatment dropout, treatment dropout and loss to follow up, reflecting four consecutive time points (about 8 weeks postpartum, about 10 weeks postpartum, between 10 and 16 weeks postpartum, and after 16 weeks postpartum respectively), for all eligible participants in a longitudinal RCT.

Participants

The current study is a secondary analysis of an RCT for women with negative birth experiences, recruited in routine public health care. Approximately 17,000 women gave birth at Uppsala university Hospital between September 2013 and February 2018, and most of them rated their overall birth experience on a Likert scale (0–10), as a standard procedure before hospital discharge. Eligible women (n = 1,523) had a negative birth experience (defined as ≤ 5 on the Likert scale), and/or an immediate caesarean section, and/or a severe postpartum haemorrhage (≥ 2,000 ml). Of 1,523 eligible women, about 20% (n = 300) gave written consent to be part of the RCT (32). The 1,523 eligible women had a mean age of 31.5 years (SD = 5.03), participants in the RCT study were 31.7 (4.6) years, and the non-participants age were 31.4 (5.1) years; the majority reported being married or having a partner (84.6 %, n = 1,291), and 50.8% (n = 775) had a university degree. Data on eligible participants are presented in Table 1.

For peer review only

Table 1.

Demographics for the eligible participants (total sample) consisting of those who participated and the non-participants.

	Total<br>*n*=1523<br>*n*(%)	Participants<br>*n*=300<br>*n*(%)	Non-participants<br>*n*=1223<br>*n*(%)
Relationship status			
Married/cohabit	1291 (95.1)	286 (21.1)	1005(74.1)
Single/other	66 (4.9)	8 (0.6)	58(4.3)
Education			
Elementary school	72(5.4)	1 (0.1)	71(5.3)
High school	489(36.6)	82 (6.1)	407(30.5)
University	775(58.0)	209 (54.2)	566(42.4)
Country of birth			
Sweden	953 (76.7)	261 (21)	692 (55.7)
Foreign born	289 (23.3)	25 (2)	264 (21.3)

Note. Missing data; *n*=166 for relationship status, *n*=187 for education, and *n*=281 for country of birth.

Sample size and power

There was no specific sample size calculation for this investigation other than the sample size estimation for the RCT (21) (power was set to 0.8 with a medium effect size) where a total sample size of 130 was needed.

Procedure

For peer review only

Women rated their birth experience as a routine measure at the hospital before discharge. Those with negative birth experiences were contacted via telephone, about eight weeks postpartum. During the telephone calls, the women were informed about the study and those interested in participating were sent study information and a consent form by post. Those who declined at this stage (*n* = 693) were asked about their reason for doing so. In total 530 eligible women did not respond to the invitation, 300 women gave written consent (participants) and 1,223 did not (non-participants). Of the 300 participants, 101 never completed baseline measures (pre-treatment dropouts). The participants who filled out the baseline questionnaires (*n* = 199) were randomized to either treatment as usual (TAU, *n* = 100) or iCBT+TAU (*n* = 99). The iCBT treatment consisted of six treatment modules including psychoeducation and interventions, with therapist support on demand, tailored for women with negative experiences of childbirth (see supplementary Table 1) (21). Regardless of treatment allocation, local health care providers in accordance with international guidelines treated all participants in the study. TAU included conventional support in accordance with the existing practices at the Department of Obstetrics and Gynecology of the participating hospital. Of the 99 allocated to treatment, a total of 41 were treatment completers (at least three of six steps completed) and 58 were treatment dropouts. All randomized participants (199) were asked to fill out questionnaires six weeks post randomization; 121 completed the follow-up measures and 78 were lost to follow-up, please see figure 1.

Figure 1 about here

Material

Based on previous knowledge about possible causes for non-participation and dropout, predictor variables were categorized into three conceptual categories (demographic, antepartum, and labour-/postpartum related variables). Obstetric data were extracted from

For peer review only - http://bmjopen.bmj.com/site/about/guidelines.xhtml

each participant's medical records and questionnaire information was taken from the U-CARE database. The Care Base Internet Platform, including its web-based part (U-CARE eService), was developed within the U-CARE program. The aim of the U-CARE research program is to prevent and reduce psychosocial malfunctioning in patients and relatives. The U-CARE eService is currently being used for interventions and data collection http://www. u-care.uu.se.

Demographic variables

Country of birth (born in Sweden /foreign-born), level of education (university/high school/elementary school), relationship status (married/partner or single/other status), and age at delivery (years).

Antepartum variables

Previous caesarean section (no/yes), counselling for fear of childbirth (no/yes), preeclampsia during pregnancy (International Classification of Disease 10th revision (ICD-10 code O14; no/yes), length of pregnancy (number of days based on second trimester ultrasound), and parity (first child/second child/third child or more).

Labour-related/postpartum variables

Mode of delivery (vaginal delivery/emergency caesarean section/immediate caesarean section/vacuum-assisted delivery/elective caesarean section), foetal presentation (vertex presentation/others), manual placenta removal (ICD-10 code O73; no/yes), epidural anaesthesia (ICD-10 code ZXH50; no/yes), intrapartum foetal distress (ICD-10 code O68; no/yes), anal sphincter injury (ICD-10 code O70; no/yes), labour dystocia (ICD-10 code O62; no/yes), severe postpartum haemorrhage (≥ 2,000 ml; no/yes), anaemia (ICD-10 code D59; no/yes), blood transfusion (ICD-10 code Z51.3; no/yes), number of children in the pregnancy (one/two or more), child transferred to neonatal intensive care unit (NICU; no/yes),

For peer review only

breastfeeding problems (ICD-10 code O92; no/yes) and overall birth experience (more severe 0-2 vs. less severe 3-5).

Dependent variables

Non-participation (*n* = 1,223) vs participation (*n*=300): eligible women who did not / did return a signed informed consent form.

Pre-treatment dropouts (*n* = 101) vs pre-treatment completers (*n*=199): women who gave written consent but did not / did proceed to complete the baseline measurements.

Treatment dropouts (*n* = 58) vs treatment completers (*n*=41): women in the iCBT arm who reported activity in 0 to 2 treatment steps of the treatment vs 3 to 6 treatment steps.

Lost to follow-up (*n* = 78) vs completed follow-up: all randomized women in either treatment arm (iCBT+TAU and TAU) who never completed the post-treatment measures vs those who completed them (*n* =121).

Statistical analysis

Logistic regression was used to determine predictors of non-participation, pre-treatment dropout, treatment dropout, and loss to follow-up. Odds ratios with 95% confidence intervals and beta values including SE are reported. Missing data were not handled. Among the predictor variables there were 3 demographics that had missing data above 5%; country of birth, education, and relationship status (18,5%, 12%, and 10,8% missing respectively). We decided not to impute these demographic missing data from antepartum, and labour-related/postpartum variables, as we think it would have resulted in arbitrary imputations. In addition, since the analyses are not multivariate but bivariate the consequences are less. All available data was used leading to slight differences regarding number of participants in the analyses. Reasons given for non-participation were categorized. SPSS version 26 was used for all analyses.

Ethics

For peer review only

The Regional Ethics Review Board in Uppsala, Sweden, approved the study (2012/495/1 and amendment 2016/11/16).

Results

Predictors of non-participation

Women with lower levels of education, multiparas and foreign-born were more often non-participants (Table 2). Women who had not been counselled for fear of childbirth, no preeclampsia during pregnancy, no sphincter injury, no intrapartum foetal distress, and those with vaginal delivery were more likely to decline participation, please see Table 2 (and supplementary Table 2 for ß,SE).

For peer review only

Table 2.
Odds ratio with 95% CI derived from logistic regression for potential predictors

	Non-participants		Pre-treatment dropout		Treatment dropout		Lost to follow up	
	N	OR 95% CI	N	OR 95% CI	N	OR 95% CI	N	OR 95% CI
Country of birth	1234	3.93(2.58-6.15)***	284	1.09(0.45-2.64)	98	1.43 (0.33-6.06)	198	1.87(0.69-5.08)
Sweden/other	953/281		259/25		89/9		181/17	
Level of education	1336		290		99		199	
University	775	1.0	208	1.0	71	1.0	148	1.0
High school	489	1.83(1.38-2.44)***	81	1.53(0.89-2.62)	27	1.32(0.53-3.28)	50	1.33(0.69-2.55)
Elementary school	72	26.2(3.62-189.9)***	1	na	1	na	1	na
Relationship status	1357		292		97		197	
married-cohabit/other	1291/66	2.06(0.97-4.37)		1.25(0.29-5.35)	95/2	na		na
Age, years	1510	0.99(0.96-1.01)	300	0.97(0.92-1.02)	99	0.95(0.86-1.05)	199	0.95(0.89-1.02)
Previous CS yes/no	1302/189	0.80(0.53-1.19)	31/264	0.78(0.36-1.68)	89/9	1.42(0.33-6.06)	177/19	0.87(0.33-2.32)
Counselling for fear of	1523		300		99		199	
childbirth, no/yes	1376/147	0.50(0.35-0.73)***	254/46	1.06(0.55-2.05)	87/12	0.68(0.18-2.52)	169/30	0.88(0.39-1.97)
Preeclampsia, no/yes	1440/83	0.59(0.36-0.96)*	276/24	1.20(0.51-2.85)	90/9	1.46(0.34-6.22)	184/15	1.39(0.48-4.00)
Pregnancy, days	1507	1.00(0.99-1.00)	299	0.99(0.98-1.01)	99	1.01(0.98-1.04)	198	1.00(0.98-1.02)
Parity	1505		299		99		198	
1st child	838	1	200	1	68	1	134	1
2nd child	448	1.65(1.22-2.22)**	71	0.97(0.55-1.73)	22	1.80(0.65-4.96)	48	1.07(0.54-2.10)
3rd child or more	219	2.15(1.40-3.30)***	28	1.52(0.68-3.40)	9	1.68(0.39-7.26)	16	1.63(0.58-4.60)
Mode of delivery	1523		300		99		199	
Vaginal delivery	783	1	129	1	40	1	82	1
Emergency CS	289	0.71(0.51-1.0)	63	1.0(0.54-1.88)	20	0.33(0.11-1.03)	40	0.74(0.34-1.58)
Immediate CS[1]	186	0.54(0.37-0.79)**	49	0.63(0.30-1.30)	19	0.19(0.06-0.63)**	36	0.42(0.18-0.99)*
Vacuum assisted	198	0.62(0.43-0.91)*	48	0.96(0.48-1.91)	15	0.29(0.84-1.01)	31	0.26(0.10-0.71)**
Elective CS	67	1.01(0.52-1.99)	11	0.17(0.02-1.41)	5	1.33(0.13-13.37)	10	2.57(0.62-10.65)
Foetal presentation	1510		300		99		199	
Vertex / other	1287/223	1.02(0.71-1.46)	256/44	0.53(0.25-1.13)	82/17	0.31(0.11-0.94)*	165/34	1.28(0.61-2.70)
Manual placenta removal	1523		300		99		199	

For peer review only

no / yes	1379/144	1.41(0.88-2.26)	278/22	0.42(0.14-1.26)	93/6	0.33(0.06-1.90)	181/18	0.99(0.36-2.66)
Epidural anaesthesia	1523		300		99		199	
no / yes	813/710	0.86(0.67-1.10)	151/149	1.12(0.69-1.80)	49/50	0.95(0.43-2.12)	102/97	1.52(0.86-2.70)
Intrapartum foetal distress	1523		300		99		199	
no / yes	1234/289	0.67(0.50-0.91)*	228/72	0.76(0.43-1.36)	71/28	0.50(0.21-1.21)	148/51	0.50(0.25-0.99)*
Anal sphincter injury	1523		300		99		199	
no / yes	1447/76	0.48(0.29-0.79)**	275/25	0.92(0.38-2.21)	88/11	1.27(0.35-4.66)	182/17	1.09(0.40-3.00)
Labour dystocia	1523		300		99		199	
no / yes	885/638	0.87(0.67-1.11)	166/134	1.26(0.78-2.04)	55/44	0.74(0.33-1.66)	114/85	0.75(0.42-1.34)
Severe haemmorhage[1]	1523		300		99		199	
no / yes	1329/194	1.19(0.80-1.76)	266/34	0.38(0.15-0.96)*	83/16	0.49(0.17-1.44)	171/28	1.00(0.44-2.28)
Anaemia	1523		300		99		199	
no / yes	1274/249	0.85(0.61-1.18)	246/54	0.57(0.29-1.12)	79/20	0.50(0.19-1.35)	158/41	0.66(0.32-1.38)
Blood transfusion	1523		300		99		199	
no / yes	1340/183	1.01(0.69-1.49)	265/35	0.65(0.29-1.45)	87/12	0.31(0.09-1.09)	173/26	0.53(0.21-1.32)
Children in the pregnancy	1508		299		99		198	
1 child / 2 children	1476/32	1.35(0.52-3.55)	294/5	0.48(0.05-4.40)	97/2	na	194/4	0.51(0.05-4.96)
Child transferred to NICU	1523		300		99		199	
no / yes	1255/268	0.83(0.60-1.14)	241/59	1.21(0.67-2.20)	78/21	0.73(0.28-1.91)	162/37	1.07(0.52-2.22)
Breastfeeding problems	1523		300		99		199	
no / yes	1505/18	0.86(0.28-2.64)	296/54	0.65(0.07-6.36)	98/1	na	196/3	0.77(0.07-8.67)
Overall birth experience[1]	1203		234		72		148	
0-2 / 3-5	305/898	1.02(0.74-1.42)	58/176	0.72(0.38-1.35)	20/52	0.27(0.09-0.79)*	40/108	0.90(0.43-1.88)

Note. The first category is the reference, for e.g. when yes/no is stated, yes is the reference category.

[1] inclusion criteria.

* p<.05, **p<.01, ***p<.001

For peer review only

Reasons why women declined to take part despite eligibility.

Of the contacted women, 693 actively declined participation and their answers were categorized into different subgroups (Table 3).

Table 3.

Reasons for non-participation (n = 693) given during telephone interview eight weeks postpartum

Reason for non-participation	N	%
Feels fine, does not need any support	326	(47)
Does not speak Swedish	134	(19)
Not interested (no further information)	77	(11)
Feels fine, has already received professional support	35	(5)
Does not feel fine, receiving/waiting for other professional support	35	(5)
Does not have the time	30	(4)
Does not have a computer	16	(2)
Not interested, will not have more kids anyway	14	(2)
Not interested, does not want to think about the delivery	13	(2)
Misunderstood the Likert scale (inclusion), had a positive experience	10	(1.4)
Not comfortable with internet/computer, prefers face-to-face therapy	3	(0.4)

Predictors of pre-treatment dropout

The only significant predictor of pre-treatment dropout was no severe postpartum haemorrhage (i.e., less than 2000 ml; Table 2).

Predictors of treatment dropout

For peer review only

For those randomized to the treatment group, dropout was significantly predicted by mode of delivery, foetal presentation, and overall birth experience (Table 2). Participants with vaginal delivery, vertex presentation and less severe overall birth experience were more likely to drop out from treatment.

Predictors of loss to follow-up

In the analyses of loss to follow-up, absence of intrapartum foetal distress and vaginal delivery (compared with immediate CS and vacuum delivery) predicted loss to follow up (Table 2). An additional analysis showed that being randomised to iCBT+TAU was a significant predictor of loss to follow up OR = 1.84 (95 % CI: 1.04-3.28), B=0.61, SE=0.29, p = .037, where 46 of 99 in iCBT+TAU and 32 of 100 in TAU were lost to follow up.

Discussion

The current study provides an explorative analysis of predictors for non-participation and drop out at different timepoints in an RCT examining iCBT for women with negative birth experiences and/or posttraumatic stress following childbirth (21). Significant predictors for non-participation and dropout were found at different stages in the recruitment process of an RCT. Women with higher education level, without previous children and those born in Sweden were more likely to enter into the study. Thereafter, women who had been counselled for fear of birth, experienced complications during the childbirth and with an overall severe birth experience were more likely to stay in the study.

A majority (80.3%) of the eligible women declined participation and our first conclusion was that a large number of those eligible did not see themselves as being in need of iCBT or wanted to take part in a clinical trial. When they were contacted by telephone during the recruitment period, the most frequent reason for declining was "I feel fine/have no

For peer review only

need of any support.” Explanations could be that the cut-off for the screening instrument was over-inclusive and/or that the other inclusion criteria (immediate caesarean section and severe postpartum haemorrhage) did not necessarily result in a negative birth experience. The content validity of the one-item Likert scale in the current trial could be discussed, as women may take different aspects of their birth experience into account. Also, the time-point for the rating could be discussed. In the current trial, all women rated their birth experience shortly after giving birth; it is difficult to determine the timepoint that would yield the most accurate rating of the birth experience. However, using a Likert scale as a tool for self-assessment of overall birth experience is well-established in clinical practice and used in research (34,35). A person’s perception of their birth experience can change over time and it is important to consider the specific timepoint used in measurement (33). Larsson et al. (36) used a VAS scale (range 1–10) for self-assessment of birth experience at two days, three months, and nine months postpartum. They found that the participants’ negative birth experiences decreased over time and suggested that use of a VAS scale was an adequate way to find women in need of follow-up after a negative birth experience.

The analysis included the inclusion criteria (immediate caesarean section, overall birth experience, and severe haemorrhage) as predictors. Non-participation was predicted by vaginal delivery vs. immediate caesarean section. Childbirth without severe haemorrhage predicted pre-treatment dropout. It is known that severe postpartum haemorrhage is a significant risk factor for developing PTSD (37,38). Treatment drop out was predicted by a less severe overall birth experience and vaginal delivery vs. immediate CS. These predictors were inclusion criteria and must therefore be interpreted with caution. However, the results might be useful for future hypothesis in further research. The three inclusion criteria in this study are experiences that potentially can have serious effects on the mental health of a birth giving woman. It may be of value to understand more about what type of care (e.g.,

For peer review only

counselling, therapy), what type of format (e.g., face to face or ICBT) and what level of support (therapist support or pure self-help) is demanded.

Three socio-demographic variables predicted non-participation: lower level of education, multiparity, and being foreign-born. Lower level of education as a greater risk for dropout is consistent with dropout in other iCBT trials (16). Multiparity was also identified as an important predictor of non-participation. The physical and psychological changes of the postpartum period are challenging for first-time mothers and they have lower levels of maternal confidence and higher levels of stress compared with multiparous women (39), which might increase their likelihood of participation. An alternative explanation is that multiparous women have less time to commit to clinical trials compared with first-time mothers. This trial's intervention addressed Swedish-speaking women; foreign-born women might see language as a barrier to participation.

Five antepartum and labour-related/postpartum variables also predicted non-participation. Women without experience of the following were more likely to be non-participants; counselling for fear of childbirth, preeclampsia, anal sphincter injury, intrapartum foetal distress, and vacuum-assisted delivery (vs. vaginal delivery). Women who had been counselled for fear of childbirth had already professionally addressed peripartum psychological problems and might therefore have been more open to support. Preeclampsia, intrapartum foetal distress, and anal sphincter injuries are all severe conditions and motivators for participation. Preeclampsia and severe postpartum haemorrhage are significant threats to the mother and may have devastating or lethal outcomes. Further, both preeclampsia and intrapartum foetal distress are potential threats to the health of the foetus, thus acting as significant stressors for the woman. Instrumental deliveries may be caused by emergency obstetric complications potentially threatening the mother or the child and are very stressful situations for the woman in their own right (30). The labour related / post-partum predictors

For peer review only

show a consistent pattern where women who did not experience these stressful events may not have had enough motivation to seek out help or support.

Pre-treatment dropout was predicted by the absence of severe haemorrhage which is mentioned above. Vertex foetal presentation (vs. other presentation) predicted treatment dropout. This is consistent with the significant predictors for non-participation where those with vertex presentation might not experience this as a stressful event enough to stay in the treatment. It might also be that those with vertex presentation who were randomized to the treatment did not find it helpful or that it did not address their problem fully in order to stay in the treatment.

Predictors for loss to follow-up were vaginal delivery vs. instrumental delivery and absence of intrapartum foetal distress. Occurrence of such events are threats to the foetus which in turn can be very stressful for the mother. Absence of these events might lead to lost interest in devoting time and energy to proceed with the follow-up assessment. Absence of immediate CS was also a significant predictor of loss to follow up and is discussed in relation to the other inclusion criteria/predictors. Finally, randomisation to iCBT+TAU (compared with TAU) was a significant predictor of lost to follow up. A majority of those who were lost to follow up from the iCBT+TAU group were also those who were treatment droputs.

Closer inspection of variables associated with non-participation and dropout can yield insights that can be used in both future research and clinical practice. Knowledge of sub-groups that are more likely to continue and complete study participation provides information about motivational factors and should be applied during the initial recruitment for similar trials. Participants where not asked why they dropped out. We believe that dropout can depend on different factors such as lack of energy and/or interest of being part of a clinical trial; dropout can also depend on the participant´s experience of not needing the intervention

For peer review only

anymore. In this trial women with lover level of education, multipara and foreign born where more often non-participants, perhaps the way of inclusion and the intervention itself must be better adapted to attract those sub-groups in the future. Translation to other languages, using simple language and pictorial material could be ways of improving adherence.

Analyses of predictors of non-participation and dropout are important for evaluating the efficacy of the interventions (6). This explorative study found predictors of non-participation and dropout that should be taken into account in future development of similar interventions. Awareness of characteristics among women who drop out and those who continue, and complete interventions is important and should get more attention during initial recruitment for similar trials.

Strengths

The current study is the first to present data on non-participation and dropouts in iCBT for women with negative birth experiences and/or posttraumatic stress following childbirth. The main strength of this study was the size of the sample and the routine public health care setting as well as consecutive recruitment. All women who gave birth were asked to rate their birth experience on a self-assessed Likert-scale and all women with a low rating were invited. This process increased the likelihood of the results being generalizable to similar clinical contexts. The exploration of dropout predictors from a large cluster of demographic variables and medical/clinical characteristics was another strength. A third strength was that reasons for dropout were explored at different stages in the study process, which allowed analysis of specific timepoints when participants were more likely to end their participation. Analyses of different timepoints for dropout could simplify the analyses of underlying reasons for withdrawal (9).

Limitations

Our study has several limitations that should be noted. Psychological problems and/or treatment-related variables were not available for analyses. Such variables are likely to be strong predictors of non-participation and dropout (9) and should be integrated in future studies. Neither discomfort with the internet or computers were analysed as factors for non-participation or dropout in the current trial. The impact of computer-related factors on adherence has been described previously (17). Further, recruitment to the study was before discharge from the hospital when the experience of birth is fresh. Thus, the eligible sample might have been different if we had asked them at a later time point. However, the assessment of the birth experience was in close conjunction to immediate CS and severe haemorrhage (the two other inclusion criteria). In some analyses there might have been a lack of power, due to the varying N, that prevented significant predictors to be found.

Conclusions

In this sample, drawn from a large population, predictors were found for non-participation and dropout at different stages in the recruitment process and during the study of an RCT. In summary, both demographic and obstetrical variables are important to attend to for both clinical and research purposes, while designing procedures to maximize participation in iCBT for postpartum women. First time mothers with high level of education and those who had adverse obstetric experiences were more likely to join and stay in the internet intervention.

Conflicts of Interest

All authors of this manuscript declare No known competing interest.

For peer review only

Authorship Contribution statement

All listed authors fulfil ICMJE authorship criteria. Author abbreviations are found in brackets after type of contribution. Writing - original draft (JS), Writing - review & editing (JS, ASS, FV, ML, ISP, AS, MJ, TP), Conceptualization (ASS, ML, ISP, AS, MJ, TP), Methodology (ASS, ML, ISP, AS, MJ, TP), Formal analysis (JS, ASS, FV, AS, TP), Investigation (JS, ASS, FV, ML, MJ), Data curation (JS, ASS, FV, ML, ISP, AS, MJ, TP), Supervision (ASS, ML, TP), Funding acquisition (ASS, MJ), and Project administration (ASS, ML, ISP, MJ). TP is the guarantor.

Transparency The manuscript's guarantor affirms that the manuscript is an honest, accurate, and transparent account of the study being reported; that no important aspects of the study have been omitted; and that any discrepancies from the study as originally planned have been explained.

Funding. This project was funded by The Regional Research Council (Regionala Forskningsrådet, RFR) grants nr 368901, 308451, and 480141; http://www.researchweb.org/ is/sverige) and Swedish research council funding for clinical research in medicine (ALF) grants nr N/A.

Role of the funding sources The funders of the study had no influence on the study design; in the collection, analysis, and interpretation of data; in the writing of the report; and in the decision to submit the article for publication. All authors confirm the independence from funders and all authors, external and internal, had full access to all of the data (including statistical reports and tables) in the study and take responsibility for the integrity of the data and the accuracy of the data analysis.

Data sharing is available on reasonable request from researchers who provide a methodologically sound proposal. Individual participant data that underlie the results

For peer review only

reported in this article, after deidentification will be shared. Proposals should be directed to agneta.skoog_svanberg@kbh.uu.se . To gain access, data requestors will need to sign a data access agreement.

Ethics statement The Regional Ethics Review Board in Uppsala, Sweden, approved the study (2012/495/1 and amendment 2016/11/16).

Acknowledgements

We would like to thank statistician Per Wikman for help with structuring the database.

Abbreviations

ICD-10: International Classification of Disease 10th revision

iCBT: Internet-based cognitive behavior therapy

RCT: Randomized controlled trial

TAU: Treatment as usual

For peer review only

References

1. Andersson G. Internet-Delivered Psychological Treatments. Annu Rev Clin Psychol. 2016 Mar 28;12(1):157–79.
2. Spek V, Cuijpers P, NyklíCek I, Riper H, Keyzer J, Pop V. Internet-based cognitive behaviour therapy for symptoms of depression and anxiety: a meta-analysis. Psychol Med. 2007 Mar;37(03):319.
3. Andrews G, Basu A, Cuijpers P, Craske MG, McEvoy P, English CL, et al. Computer therapy for the anxiety and depression disorders is effective, acceptable and practical health care: An updated meta-analysis. J Anxiety Disord. 2018 Apr;55:70–8.
4. Cuijpers P, Donker T, van Straten A, Li J, Andersson G. Is guided self-help as effective as face-to-face psychotherapy for depression and anxiety disorders? A systematic review and meta-analysis of comparative outcome studies. Psychol Med. 2010 Dec;40(12):1943–57.
5. Christensen H, Griffiths KM, Farrer L. Adherence in Internet Interventions for Anxiety and Depression. J Med Internet Res. 2009 Apr 24;11(2):e13.
6. Donkin L, Christensen H, Naismith SL, Neal B, Hickie IB, Glozier N. A Systematic Review of the Impact of Adherence on the Effectiveness of e-Therapies. J Med Internet Res. 2011 Aug 5;13(3):e52.
7. Kiluk BD, Sugarman DE, Nich C, Gibbons CJ, Martino S, Rounsaville BJ, et al. A Methodological Analysis of Randomized Clinical Trials of Computer-Assisted Therapies for Psychiatric Disorders: Toward Improved Standards for an Emerging Field. Am J Psychiatry. 2011 Aug;168(8):790–9.
8. Baumeister H, Reichler L, Munzinger M, Lin J. The impact of guidance on Internet-based mental health interventions — A systematic review. Internet Interv. 2014 Oct;1(4):205–15.
9. Melville KM, Casey LM, Kavanagh DJ. Dropout from Internet-based treatment for psychological disorders. Br J Clin Psychol. 2010 Nov;49(4):455–71.
10. Richards D, Richardson T. Computer-based psychological treatments for depression: A systematic review and meta-analysis. Clin Psychol Rev. 2012 Jun;32(4):329–42.
11. Kuester A, Niemeyer H, Knaevelsrud C. Internet-based interventions for posttraumatic stress: A meta-analysis of randomized controlled trials. Clin Psychol Rev. 2016 Feb;43:1–16.
12. Melville KM, Casey LM, Kavanagh DJ. Psychological treatment dropout among pathological gamblers. Clin Psychol Rev. 2007 Dec;27(8):944–58.

For peer review only

13. Sieverink F, Kelders SM, van Gemert-Pijnen JE. Clarifying the Concept of Adherence to eHealth Technology: Systematic Review on When Usage Becomes Adherence. J Med Internet Res. 2017 Dec 6;19(12):e402.
14. Eysenbach G. The Law of Attrition. J Med Internet Res. 2005 Mar 31;7(1):e11.
15. Gul RB, Ali PA. Clinical trials: the challenge of recruitment and retention of participants. J Clin Nurs. 2010 Jan;19(1–2):227–33.
16. Karyotaki E, Kleiboer A, Smit F, Turner DT, Pastor AM, Andersson G, et al. Predictors of treatment dropout in self-guided web-based interventions for depression: an 'individual patient data' meta-analysis. Psychol Med. 2015 Oct;45(13):2717–26.
17. Beatty L, Binnion C. A Systematic Review of Predictors of, and Reasons for, Adherence to Online Psychological Interventions. Int J Behav Med. 2016 Dec;23(6):776–94.
18. Edmonds M, Hadjistavropoulos HD, Schneider LH, Dear BF, Titov N. Who benefits most from therapist-assisted internet-delivered cognitive behaviour therapy in clinical practice? Predictors of symptom change and dropout. J Anxiety Disord. 2018 Mar;54:24–32.
19. Hedman E, Ljótsson B, Kaldo V, Hesser H, El Alaoui S, Kraepelien M, et al. Effectiveness of Internet-based cognitive behaviour therapy for depression in routine psychiatric care. J Affect Disord. 2014 Feb;155:49–58.
20. Knaevelsrud C, Maercker A. Internet-based treatment for PTSD reduces distress and facilitates the development of a strong therapeutic alliance: a randomized controlled clinical trial. BMC Psychiatry. 2007;7(1):13.
21. Crawley R, Ayers S, Button S, Thornton A, Field AP, Lee S, et al. Feasibility and acceptability of expressive writing with postpartum women: a randomised controlled trial. BMC Pregnancy Childbirth [Internet]. 2018 Dec;18(1).
22. Socialstyrelsen. Statistik om graviditeter, förlossningar och nyfödda barn 2019 [Internet]. Stockholm; 2020 [cited 2021 Sep 8] p. 4. Report No.: Art.nr: 2020-12-7051. Available from: https://www.socialstyrelsen.se/globalassets/sharepoint-dokument/artikelkatalog/statistik/2020-12-7051.pdf
23. de Graaff LF, Honig A, van Pampus MG, Stramrood CAI. Preventing post-traumatic stress disorder following childbirth and traumatic birth experiences: a systematic review. Acta Obstet Gynecol Scand. 2018 Jun;97(6):648–56.
24. Haagen JFG, Moerbeek M, Olde E, van der Hart O, Kleber RJ. PTSD after childbirth: A predictive ethological model for symptom development. J Affect Disord. 2015 Oct;185:135–43.

For peer review only

25. Alcorn KL, O'Donovan A, Patrick JC, Creedy D, Devilly GJ. A prospective longitudinal study of the prevalence of post-traumatic stress disorder resulting from childbirth events. Psychol Med. 2010 Nov;40(11):1849–59.

26. Wijma K, Söderquist J, Wijma B. Posttraumatic stress disorder after childbirth: A cross sectional study. J Anxiety Disord. 1997 Nov;11(6):587–97.

27. Olde E, van der Hart O, Kleber RJ, van Son MJM, Wijnen HAA, Pop VJM. Peritraumatic Dissociation and Emotions as Predictors of PTSD Symptoms Following Childbirth. J Trauma Dissociation. 2005 Sep 13;6(3):125–42.

28. Garthus-Niegel S, von Soest T, Vollrath ME, Eberhard-Gran M. The impact of subjective birth experiences on post-traumatic stress symptoms: a longitudinal study. Arch Womens Ment Health. 2013 Feb;16(1):1–10.

29. Dekel S, Stuebe C, Dishy G. Childbirth Induced Posttraumatic Stress Syndrome: A Systematic Review of Prevalence and Risk Factors. Front Psychol. 2017 Apr 11;8.

30. Grekin R, O'Hara MW. Prevalence and risk factors of postpartum posttraumatic stress disorder: A meta-analysis. Clin Psychol Rev. 2014 Jul;34(5):389–401.

31. Yildiz PD, Ayers S, Phillips L. The prevalence of posttraumatic stress disorder in pregnancy and after birth: A systematic review and meta-analysis. J Affect Disord. 2017 Jan;208:634–45.

32. Sjömark J, Parling T, Jonsson M, Larsson M, Skoog Svanberg A. A longitudinal, multi-centre, superiority, randomized controlled trial of internet-based cognitive behavioural therapy (iCBT) versus treatment-as-usual (TAU) for negative experiences and posttraumatic stress following childbirth: the JUNO study protocol. BMC Pregnancy Childbirth. 2018;18(387).

33. Elm E von, Altman D, Egger M, Pocock S, Gotzsche P, Vandenbroucke J. The Strengthening the Reporting of Observational Studies in Epidemiology (STROBE) Statement: guidelines for reporting observational studies. https://www.equator-network.org/reporting-guidelines/strobe/

34. Waldenström U. Why do some women change their opinion about childbirth over time? Birth. 2004;31(2):102–7.

35. Waldenström U, Hildingsson I, Rubertsson C, Raadestad I. A negative birth experience: prevalence and risk factors in a national sample. Birth. 2004;31(1):17–27.

36. Larsson C, Saltvedt S, Edman G, Wiklund I, Andolf E. Factors independently related to a negative birth experience in first-time mothers. Sex Reprod Healthc. 2011 Apr;2(2):83–9.

37. van Steijn ME, Scheepstra KWF, Zaat TR, van Rooijen DE, Stramrood CAI, Dijksman

For peer review only

LM, et al. Severe postpartum hemorrhage increases risk of posttraumatic stress disorder: a prospective cohort study. J Psychosom Obstet Gynecol. 2020 Mar 17;1–11.

38. Michelet D, Ricbourg A, Gosme C, Rossignol M, Schurando P, Barranger E, et al. Emergency hysterectomy for life-threatening postpartum haemorrhage: Risk factors and psychological impact. Gynécologie Obstétrique Fertil. 2015 Dec;43(12):773–9.

39. Kristensen IH, Simonsen M, Trillingsgaard T, Pontoppidan M, Kronborg H. First-time mothers' confidence mood and stress in the first months postpartum. A cohort study. Sex Reprod Healthc. 2018 Oct;17:43–9.

Figure caption.

Figure 1. Flowchart

For peer review only

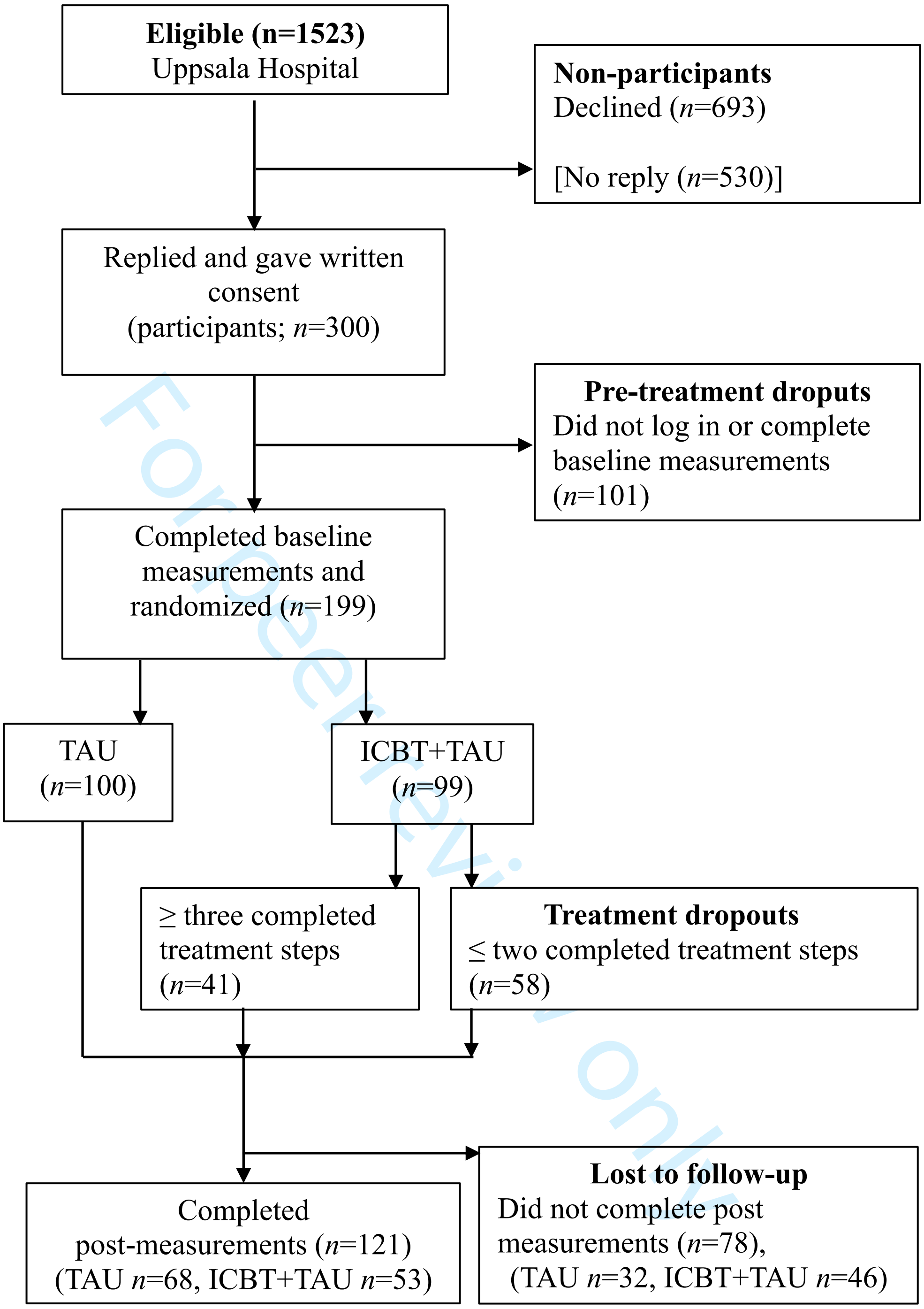

Figure 1. Flowchart

Supplementary Table 1.

Week by week content of the iCBT treatment

Week	Content
1st	Information, psychoeducation, breathing retraining
2nd	Vignettes, common symptoms, fear and avoidance
3rd	Depressive symptoms, significance of relationships, “reflective listening”
4th	Exposure, talking about the childbirth
5th	Managing anxiety and depressive symptoms, psychological health, values, recovery
6th	Summary, repetition and relapse prevention

Note. Every week contained homework assignments based on the content of the module

For peer review only

Supplementary Table 2.
Odds ratio with 95% CI, Beta values, and SE derived from logistic regression, for potential predictors

	Non-participants		Pre-treatment dropout		Treatment dropout		Lost to follow up	
	N	OR 95% CI<br>*B*, S.E	N	OR 95% CI<br>*B*, S.E	N	OR 95% CI<br>*B*, S.E	N	OR 95% CI<br>*B*, S.E
Country of birth<br>Sweden/other	1234<br>953/281	3.93(2.58-6.15)***<br>1.38, 0.22	284<br>259/25	1.09(0.45-2.64)<br>0.09, 0.45	98<br>89/9	1.43 (0.33-6.06)<br>0.35, 0.74	198<br>181/17	1.87(0.69-5.08)<br>0.63, 0.51
Level of education	1336		290		99		199	
University	775	1.0	208	1.0	71	1.0	148	1.0
High school	489	1.83(1.38-2.44)***<br>0.61, 0.15	81	1.53(0.89-2.62)<br>0.43, 0.28	27	1.32(0.53-3.28)<br>0.28, 0.46	50	1.33(0.69-2.55)<br>0.28, 0.33
Elementary school	72	26.2(3.62-189.9)***<br>3.27, 1.01	1	na	1	na	1	na
Relationship status<br>married-cohabit/other	1357<br>1291/66	2.06(0.97-4.37)<br>0.72, 0.38	292	1.25(0.29-5.35)<br>0.256, 0.74	97<br>95/2	na	197<br>192/5	na
Age<br>years	1510	0.99(0.96-1.01)<br>-0.12, 0.013	300	0.97(0.92-1.02)<br>-0.034, 0.86	99	0.95(0.86-1.05)<br>-0.52, 0.05	199	0.95(0.89-1.02)<br>-0.048, 0.03
Previous CS<br>no / yes	1491<br>1302/189	0.80(0.53-1.19)<br>-0.23, 0.21	295<br>31/264	0.78(0.36-1.68)<br>-0.25, 0.39	98<br>89/9	1.42(0.33-6.06)<br>0.35, 0.74	196<br>177/19	0.87(0.33-2.32)<br>-0.14, 0.50
Counselling for fear of childbirth, no / yes	1523<br>1376/147	0.50(0.35-0.73)***<br>-0.69, 0.19	300<br>254/46	1.06(0.55-2.05)<br>0.06, 034	99<br>87/12	0.68(0.18-2.52)<br>-0.40, 0.62	199<br>169/30	0.88(0.39-1.97)<br>-0.13, 0.41
Preeclampsia<br>no / yes	1523<br>1440/83	0.59(0.36-0.96)*<br>-0.53, 0.25	300<br>276/24	1.20(0.51-2.85)<br>0.18, 0.44	99<br>90/9	1.46(0.34-6.22)<br>0.38, 0.74	199<br>184/15	1.39(0.48-4.00)<br>0.33, 0.54
Length of pregnancy<br>days	1507	1.00(0.99-1.00)<br>-0.003, 0.004	299	0.99(0.98-1.01)<br>-0.008, 0.008	99	1.01(0.98-1.04)<br>0.01, 0.013	198	1.00(0.98-1.02)<br>0.002, 0.01
Parity	1505		299		99		198	
1st child	838	1	200	1	68	1	134	1

For peer review only

2nd child	448	1.65(1.22-2.22)**<br>0.50, 0.15	71	0.97(0.55-1.73)<br>-0.28, 029	22	1.80(0.65-4.96)<br>0.58, 0.52	48	1.07(0.54-2.10)<br>0.06, 0.34
3rd child or more	219	2.15(1.40-3.30)***<br>0.77, 0.081	28	1.52(0.68-3.40)<br>0.42, 0.41	9	1.68(0.39-7.26)<br>0.52, 0.75	16	1.63(0.58-4.60)<br>0.49, 0.53
Mode of delivery	1523		300		99		199	
Vaginal delivery	783	1	129	1	40	1	82	1
Emergency CS	289	0.71(0.51-1.0)<br>-0.34, 0.17	63	1.0(0.54-1.88)<br>0.003, 0.32	20	0.33(0.11-1.03)<br>-1.1, 0.58	40	0.74(0.34-1.58)<br>-0.31, 0.39
Immediate CS[1]	186	0.54(0.37-0.79)**<br>-0.61, 0.19	49	0.63(0.30-1.30)<br>-0.46, 0.37	19	0.19(0.06-0.63)**<br>-1.64, 0.60	36	0.42(0.18-0.99)*<br>-0.86, 0.43
Vacuum assisted	198	0.62(0.43-0.91)*<br>-0.475, 0.19	48	0.96(0.48-1.91)<br>-0.04, 0.35	15	0.29(0.84-1.01)<br>-1.23, 0.63	31	0.26(0.10-0.71)**<br>-1.33, 0.51
Elective CS	67	1.01(0.52-1.99)<br>0.01, 0.34	11	0.17(0.02-1.41)<br>-1.75, 1.06	5	1.33(0.13-13.37)<br>0.29, 1.18	10	2.57(0.62-10.65)<br>0.95, 0.73
Foetal presentation	1510	1.02(0.71-1.46)	300	0.53(0.25-1.13)	99	0.31(0.11-0.94)*	199	1.28(0.61-2.70)
Vertex / other	1287/223	0.20, 0.18	256/44	-0.63, 0.38	82/17	-1.16, 0.56	165/34	0.24, 0.38
Manual placenta removal	1523	1.41(0.88-2.26)	300	0.42(0.14-1.26)	99	0.33(0.06-1.90)	199	0.99(0.36-2.66)
no / yes	1379/144	0.346, 0.24	278/22	-0.88, 0.57	93/6	-1.11, 0.89	181/18	-0.014, 0.51
Epidural anaesthesia	1523	0.86(0.67-1.10)	300	1.12(0.69-1.80)	99	0.95(0.43-2.12)	199	1.52(0.86-2.70)
no / yes	813/710	-0.15, 0.13	151/149	0.11, 024	49/50	-0.049, 0.41	102/97	0.42, 0.29
Intrapartum foetal distress	1523	0.67(0.50-0.91)*	300	0.76(0.43-1.36)	99	0.50(0.21-1.21)	199	0.50(0.25-0.99)*
no / yes	1234/289	-0.39, 0.15	228/72	-0.27, 0.29	71/28	-0.69, 0.45	148/51	-0.70, 0.36
Anal sphincter injury	1523	0.48(0.29-0.79)**	300	0.92(0.38-2.21)	99	1.27(0.35-4.66)	199	1.09(0.40-3.00)
no / yes	1447/76	-0.73, 0.25	275/25	-0.82, 045	88/11	0.24, 0.66	182/17	0.09, 0.52
Labour dystocia	1523	0.87(0.67-1.11)	300	1.26(0.78-2.04)	99	0.74(0.33-1.66)	199	0.75(0.42-1.34)
no / yes	885/638	-0.14, 0.13	166/134	0.23, 0.25	55/44	-0.3, 0.41	114/85	-0.29, 0.30
Severe haemmorhage[1]	1523	1.19(0.80-1.76)	300	0.38(0.15-0.96)*	99	0.49(0.17-1.44)	199	1.00(0.44-2.28)

For peer review only

no / yes	1329/194	0.17, 0.20	266/34	-0.95, 0.47	83/16	-0.72, 0.55	171/28	0.004, 0.42
Anaemia	1523	0.85(0.61-1.18)	300	0.57(0.29-1.12)	99	0.50(0.19-1.35)	199	0.66(0.32-1.38)
no / yes	1274/249	-0.16, 0.17	246/54	-0.56, 0.35	79/20	-0.69, 0.51	158/41	-0.41, 0.37
Blood transfusion	1523	1.01(0.69-1.49)	300	0.65(0.29-1.45)	99	0.31(0.09-1.09)	199	0.53(0.21-1.32)
no / yes	1340/183	0.011, 0.20	265/35	-0.43, 0.41	87/12	-1.19, 0.65	173/26	-0.64, 0.47
Children in the pregnancy	1508	1.35(0.52-3.55)	299	0.48(0.05-4.40)	99	na	198	0.51(0.05-4.96)
1 child / 2 children	1476/32	0.30, 0.49	294/5	-0.72, 1.12	97/2		194/4	-0.68, 1.16
Child transferred to NICU	1523	0.83(0.60-1.14)	300	1.21(0.67-2.20)	99	0.73(0.28-1.91)	199	1.07(0.52-2.22)
no / yes	1255/268	-0.19, 0.16	241/59	0.20, 0.30	78/21	-0.32, 0.49	162/37	0.069, 0.37
Breastfeeding problems	1523	0.86(0.28-2.64)	300	0.65(0.07-6.36)	99	na	199	0.77(0.07-8.67)
no / yes	1505/18	-0.15, 0.57	296/54	-0.43, 1.16	98/1		196/3	-0.26, 1.23
Overall birth experience[1]	1203	1.02(0.74-1.42)	234	0.72(0.38-1.35)	72	0.27(0.09-0.79)*	148	0.90(0.43-1.88)
0-2 / 3-5	305/898	0.023, 0.17	58/176	-0.34, 0.32	20/52	-1.31, 0.55	40/108	-0.11, 0.38

Note. The first category is the reference, for e.g. when yes/no is stated, yes is the reference category.

[1] inclusion criteria.

* p<.05, **p<.01, ***p<.001

For peer review only

Reporting checklist for cohort study.

Based on the STROBE cohort guidelines.

Instructions to authors

Complete this checklist by entering the page numbers from your manuscript where readers will find each of the items listed below.

Your article may not currently address all the items on the checklist. Please modify your text to include the missing information. If you are certain that an item does not apply, please write "n/a" and provide a short explanation.

Upload your completed checklist as an extra file when you submit to a journal.

In your methods section, say that you used the STROBE cohortreporting guidelines, and cite them as:

von Elm E, Altman DG, Egger M, Pocock SJ, Gotzsche PC, Vandenbroucke JP. The Strengthening the Reporting of Observational Studies in Epidemiology (STROBE) Statement: guidelines for reporting observational studies.

		Reporting Item	Page Number
**Title and abstract**		Internet-based Cognitive Behavior Therapy (iCBT) for women with negative birth experiences and/or posttraumatic stress following childbirth: Prevalence and predictors of non-participation and dropout	
Title	#1a	Indicate the study's design with a commonly used term in the title or the abstract	Title page
Abstract	#1b	Provide in the abstract an informative and balanced summary of what was done and what was found	Abstract page
**Introduction**			
Background / rationale	#2	Explain the scientific background and rationale for the investigation being reported	5-7
Objectives	#3	State specific objectives, including any prespecified hypotheses	7
**Methods**			
Study design	#4	Present key elements of study design early in the paper	8

For peer review only

Setting	#5	Describe the setting, locations, and relevant dates, including periods of recruitment, exposure, follow-up, and data collection	8
Eligibility criteria	#6a	Give the eligibility criteria, and the sources and methods of selection of participants. Describe methods of follow-up.	8-10
Eligibility criteria	#6b	For matched studies, give matching criteria and number of exposed and unexposed	n.a
Variables	#7	Clearly define all outcomes, exposures, predictors, potential confounders, and effect modifiers. Give diagnostic criteria, if applicable	10-12
Data sources / measurement	#8	For each variable of interest give sources of data and details of methods of assessment (measurement). Describe comparability of assessment methods if there is more than one group. Give information separately for for exposed and unexposed groups if applicable.	10-12
Bias	#9	Describe any efforts to address potential sources of bias	18-23
Study size	#10	Explain how the study size was arrived at	9
Quantitative variables	#11	Explain how quantitative variables were handled in the analyses. If applicable, describe which groupings were chosen, and why	10-12
Statistical methods	#12a	Describe all statistical methods, including those used to control for confounding	12
Statistical methods	#12b	Describe any methods used to examine subgroups and interactions	n.a.
Statistical methods	#12c	Explain how missing data were addressed	12
Statistical methods	#12d	If applicable, explain how loss to follow-up was addressed	12
Statistical methods	#12e	Describe any sensitivity analyses	n.a
**Results**			
Participants	#13a	Report numbers of individuals at each stage of study—eg numbers potentially eligible, examined for eligibility, confirmed eligible, included in the study, completing follow-up, and analysed. Give	fig 1, table 2

For peer review only

		information separately for for exposed and unexposed groups if applicable.	
Participants	#13b	Give reasons for non-participation at each stage	Fig 1
Participants	#13c	Consider use of a flow diagram	Fig 1
Descriptive data	#14a	Give characteristics of study participants (eg demographic, clinical, social) and information on exposures and potential confounders. Give information separately for exposed and unexposed groups if applicable.	Table 1
Descriptive data	#14b	Indicate number of participants with missing data for each variable of interest	Table 2
Descriptive data	#14c	Summarise follow-up time (eg, average and total amount)	10
Outcome data	#15	Report numbers of outcome events or summary measures over time. Give information separately for exposed and unexposed groups if applicable.	Table 2
Main results	#16a	Give unadjusted estimates and, if applicable, confounder-adjusted estimates and their precision (eg, 95% confidence interval). Make clear which confounders were adjusted for and why they were included	Table 2
Main results	#16b	Report category boundaries when continuous variables were categorized	Table 2
Main results	#16c	If relevant, consider translating estimates of relative risk into absolute risk for a meaningful time period	n.a
Other analyses	#17	Report other analyses done—eg analyses of subgroups and interactions, and sensitivity analyses	n.a
**Discussion**			

For peer review only

Key results	#18	Summarise key results with reference to study objectives	18-22
Limitations	#19	Discuss limitations of the study, taking into account sources of potential bias or imprecision. Discuss both direction and magnitude of any potential bias.	22-23
Interpretation	#20	Give a cautious overall interpretation considering objectives, limitations, multiplicity of analyses, results from similar studies, and other relevant evidence.	18-23
Generalisability	#21	Discuss the generalisability (external validity) of the study results	18-23
**Other Information**			
Funding	#22	Give the source of funding and the role of the funders for the present study and, if applicable, for the original study on which the present article is based	24

None The STROBE checklist is distributed under the terms of the Creative Commons Attribution License CC-BY. This checklist can be completed online using https://www.goodreports.org/, a tool made by the EQUATOR Network in collaboration with Penelope.ai

For peer review only
